# Radiotherapy Reprograms CCR8⁺ Regulatory T Cells via REL-NF-κB to Enforce Spatial Immune Exclusion in Hepatocellular Carcinoma

**DOI:** 10.7150/ijbs.136364

**Published:** 2026-09-24

**Authors:** Huanliang Chen, Shuxuan Wang, Bufu Tang, Yanyan Lin, Pan Zhou, Jiaoyang Yang, Qian-Qian Zhao, Zhao-Chong Zeng

**Affiliations:** 1 Department of Radiation Oncology, Zhongshan Hospital, Fudan University, Shanghai, 200032, China.; 2 Zhongshan Hospital, Fudan University, Shanghai, 200032, China.; 3 Department of Interventional Radiology, Zhongshan Hospital, Fudan University, Shanghai, 200032, China.

**Keywords:** CCR8, Tregs, immunotherapy, hepatocellular carcinoma

## Abstract

Radiotherapy is increasingly incorporated into the treatment of hepatocellular carcinoma (HCC), but adaptive immunosuppressive remodeling of the tumor microenvironment can limit efficacy. We integrated public bulk, single-cell, and spatial transcriptomic cohorts with a murine single-cell atlas, paired human radiotherapy specimens, and functional murine studies. High intratumoral regulatory T cell (Treg) infiltration was associated with poor prognosis across two cohorts. Analyses of single cells identified a CCR8⁺ Treg state that was enriched in advanced tumors, exhibited enhanced suppressive features, and was conserved across species. Spatial analyses showed that CCR8⁺ Tregs accumulated in peritumoral regions enriched in myeloid cells and fibrotic stroma, where CD8⁺ T cell infiltration was limited. Hypofractionated radiotherapy expanded suppressive CCR8⁺ Tregs, while radiation enhanced CCL1 secretion by M2-like tumor-associated macrophages (TAMs), providing a recruitment signal. Mechanistically, REL knockdown, chromatin immunoprecipitation, and promoter reporter assays demonstrated that radiation activates REL, which directly binds to and transactivates the CCR8 promoter. CCR8 blockade enhanced radiotherapy efficacy, reduced suppressive Treg features, and increased cytotoxic CD8⁺ T cell activity. These findings identify a REL-NF-κB-CCR8 axis as a mediator of immune adaptation following radiotherapy and support combination therapies targeting CCR8 in HCC.

## Introduction

Hepatocellular carcinoma (HCC) remains a leading cause of mortality related to cancer worldwide 1,2. Because of its insidious onset, many patients are diagnosed at advanced stages and are no longer eligible for curative surgery 3,4. In this setting, radiotherapy, particularly stereotactic body radiation therapy, has become an important option for local tumor control 5,6. However, its clinical benefit is often limited by intrinsic or acquired radioresistance, leading to recurrence and poor outcome 7. Increasing evidence suggests that this resistance arises not only from DNA damage responses within tumor cells, but also from dynamic remodeling of the tumor microenvironment (TME) following irradiation 8.

Among the immune mechanisms implicated in this process, Tregs have emerged as key mediators of immunosuppression induced by radiotherapy 9,10. Although ionizing radiation can promote immunogenic cell death and stimulate antitumor immunity 11,12, it may also enhance the recruitment and activation of Tregs within tumors, thereby limiting durable therapeutic efficacy 7-10. While strategies targeting pan-Treg markers such as CD25 have been explored, their clinical utility remains constrained by limited selectivity and the risk of systemic immune toxicity 13.

CCR8 has recently emerged as a more selective marker of Tregs that infiltrate tumors and exhibit potent immunosuppressive activity 14,15. Compared with conventional Treg markers, CCR8 is preferentially enriched in activated intratumoral Tregs and is minimally expressed in peripheral immune compartments 14-16. In addition, CCR8 and CCL1 form a chemokine receptor and ligand pair that promotes Treg recruitment and supports their immunosuppressive function within the TME 14,15,17,18. Targeting CCR8⁺ Tregs can enhance antitumor immunity and improve responses to immune checkpoint blockade 14,16. However, whether CCR8⁺ Tregs limit radiotherapy efficacy in HCC, how radiotherapy shapes this population and its spatial organization, and whether targeting this axis can improve radiotherapy efficacy remain unclear.

In this study, we combined reanalysis of public bulk, single-cell, and spatial transcriptomic datasets with newly generated murine single-cell profiling, paired human radiotherapy specimens, and functional experiments to investigate the role of CCR8⁺ Tregs in the response to radiotherapy. We show that radiotherapy engages a REL-NF-κB-CCR8 axis that expands a spatially restrictive, hypersuppressive Treg state, and we provide direct experimental evidence that REL transcriptionally controls CCR8. Distinct from prior work that used CCR8 mainly as a depletion target 16 or targeted pan-Treg markers such as CD25 13, our findings place CCR8⁺ Tregs within an integrated framework of radiotherapy response, spatial immune exclusion, and direct transcriptional regulation, and identify CCR8 as a mechanistically relevant and therapeutically actionable target for enhancing radiotherapy efficacy in HCC.

## Materials and Methods

Detailed information is provided in the Supplementary Materials and Methods.

### Data collection

Bulk RNA-seq data and clinicopathological annotations were obtained from TCGA-LIHC (via UCSC Xena) 19 and, as an independent validation cohort, ICGC-LIHC (ICGC Data Portal) 20. Human HCC single-cell RNA sequencing (scRNA-seq) data (GSE149614) 21 and Visium spatial transcriptomic data (GSE245908) 22 were retrieved from public repositories. A published HCC T cell scRNA-seq dataset (GSE98638) was used to derive the Treg-specific gene signature (Table S2). In-house murine scRNA-seq libraries were generated using the 10x Genomics Chromium system.

### Patients and tissue samples

Human HCC tissue samples (n = 12 for FOXP3 immunohistochemistry, Table S1; n = 4 paired pre- and post-radiotherapy specimens for multiplex immunofluorescence, Table S3) were obtained with approval from the Institutional Ethics Committee of Zhongshan Hospital, Fudan University, and with written informed consent (No. B2026-189). Radiotherapy response was assessed by mRECIST.

### Cell culture and primary cell isolation

Hepa1-6 and H22 murine hepatoma lines were maintained in DMEM or RPMI-1640 containing 10% FBS and 1% penicillin-streptomycin. Conditioned medium from H22 cells was collected when cultures reached approximately 80% confluence, centrifuged at 300 × g for 10 min, and passed through a 0.22 μm filter. Primary CD8⁺ T cells and CD4⁺CD25⁺ Tregs were enriched from C57BL/6 spleens by magnetic-activated cell sorting (MACS), and Treg purity was confirmed by flow cytometry (Figure S2K). Bone marrow-derived macrophages (BMDMs) were differentiated with M-CSF for 7 days and polarized to M2-like TAMs with IL-4 and IL-13 (Figure S4A-B).

### Immunohistochemistry and multiplex immunofluorescence

Tissue sections embedded in paraffin were processed for FOXP3 immunohistochemistry using DAB and multiplex immunofluorescence using tyramide signal amplification, followed by DAPI counterstaining.

### Treg suppression and chemotaxis assays

For suppression assays, CD8⁺ T cells labeled with CFSE were cocultured with Tregs in the presence of anti-CD3 and anti-CD28 antibodies. Proliferation, including the percentage of divided cells and the division index, and IFN-γ production were quantified by flow cytometry. For chemotaxis assays, Tregs isolated by MACS were seeded into Transwell inserts, with basal medium, recombinant CCL1, or M2-like TAM-conditioned medium placed in the lower chamber. Migrated cells were quantified by flow cytometry using a defined acquisition time.

### Flow cytometry

Single-cell suspensions were stained with a Fixable Viability Dye, Fc-blocked, and stained for surface and intracellular markers.

### Murine tumor models and treatment

Hepa1-6 cells (subcutaneous) or Hepa1-6-luc cells (intrahepatic) were inoculated into male C57BL/6 mice as indicated. Radiation regimens included hypofractionated radiotherapy (HFRT) and conventional radiotherapy (RT). HFRT was delivered as 6 Gy × 2 in the radiotherapy-alone experiments (Figure 3A, Figure S2A) and as 8 Gy × 3 in the combination experiments (Figure S2B), and conventional RT was delivered as 2 Gy × 8 (Figure 3A, Figure S2A). The 6 Gy × 2 and 2 Gy × 8 schedules were matched for biologically effective dose (19.2 Gy, α/β = 10 Gy). Anti-CCR8 or isotype IgG was administered intraperitoneally twice weekly. Tumor burden was monitored by caliper measurement and bioluminescence imaging, body weight was recorded as a toxicity readout, and the liver/body-weight ratio was recorded as an additional measure of intrahepatic tumor burden. All animal experiments were approved by the Institutional Animal Care and Use Committee of Zhongshan Hospital, Fudan University (No. 2024-096).

### Molecular and mechanistic assays

REL was silenced in Tregs by siRNA transfection. Chromatin immunoprecipitation (ChIP) with an anti-c-Rel antibody was performed in primary murine Tregs to assess c-Rel occupancy at the CCR8 promoter. CCR8 promoter luciferase reporter constructs containing either the unmutated sequence or mutations at the binding sites were cotransfected with a REL expression vector or an empty vector into HEK293T cells and analyzed using a luciferase assay with dual reporters. CCL1 secretion by irradiated M2-like TAMs and Hepa1-6 cells was measured in culture supernatants using ELISA. Activation of the NF-κB pathway was assessed by Western blotting (p65 and p-p65) and by flow cytometric analysis of p-c-Rel.

### Statistical analysis

Analyses were performed using R (v4.2.1) and GraphPad Prism 9.0. Appropriate parametric or nonparametric tests were selected according to the data distribution and experimental design, with corrections for multiple comparisons applied when necessary. Survival analyses were performed using Kaplan-Meier estimates, log-rank tests, and Cox proportional hazards models. All tests were two-sided. Data are presented as mean ± SD, and P < 0.05 was considered statistically significant.

## Results

### High Treg infiltration predicts poor prognosis and inferior radiotherapy response in HCC

To assess the prognostic relevance of Tregs in HCC, we analyzed the TCGA-LIHC and ICGC-LIHC cohorts. Patients with a high abundance of Tregs within tumors had significantly worse overall survival than those with low Treg infiltration in both cohorts, and the direction of this association was consistent (TCGA log-rank P = 0.0224, Figure 1A; ICGC log-rank P = 0.0497, Figure S1A). Time-dependent ROC analysis indicated that Treg abundance carried prognostic information, particularly for long-term survival (5-year AUC = 0.76 in TCGA and 0.93 in ICGC; Figure 1B, Figure S1B). Subgroup analyses showed an overall pattern supporting an association between high Treg infiltration and worse outcome across most clinical strata (Figure 1C, Figure S1C). Prognostic nomograms integrated Treg infiltration with clinical variables to estimate 1-, 3-, and 5-year overall survival (Figure 1D, Figure S1D).

To validate the clinical relevance of Tregs in the response to radiotherapy, we examined FOXP3 immunohistochemistry in tumor tissues from HCC patients treated with radiotherapy. Based on post-radiotherapy imaging response assessed by mRECIST, responders exhibited markedly lower FOXP3⁺ Treg infiltration than nonresponders (Figure 1I), supporting the association between intratumoral Treg accumulation and unfavorable therapeutic outcome.

To identify key molecular determinants associated with the Treg phenotype, we first established a candidate set of genes specific to Tregs (Table S2) and identified differentially expressed genes (DEGs) between tumor tissues and adjacent normal tissues in TCGA-LIHC (Figure 1E). We then applied two complementary machine learning approaches (XGBoost feature importance and random survival forest permutation importance), both of which ranked CCR8 among the informative features associated with Tregs (Figure 1F-G). Consensus intersection of DEGs with the two machine learning selections yielded four overlapping candidates: CCR8, TNFRSF4, TNFRSF9, and RTKN2 (Figure 1H). Among them, CCR8 was prioritized for further investigation because of its established role as the receptor for CCL1 and its preferential expression on Tregs within tumors, where it has been implicated in both Treg recruitment and immunosuppressive activity.

### CCR8⁺ Tregs constitute a key population responsible for mediating the immunosuppressive functions of Tregs

To characterize the transcriptional heterogeneity of lymphocytes infiltrating HCC tumors, we reanalyzed the GSE149614 scRNA-seq dataset 21 and mapped the major cell populations using t-SNE (Figure 2A). Unsupervised subclustering of FOXP3⁺ Tregs identified six distinct subsets (T0-T5), highlighting marked intratumoral heterogeneity (Figure 2B). Among them, the T0 cluster was preferentially enriched in tumor tissue and further accumulated in patients with advanced (stage IV) disease, suggesting a link to malignant progression (Figure 2C).

Phenotypic scoring across four functional dimensions (naive, suppressive, resident, and proliferative) showed that T0 displayed the strongest suppressive signature (Figure 2E-F). Consistently, KEGG pathway analysis demonstrated enrichment of immune regulatory pathways in T0, including allograft rejection and related antigen-recognition programs (Figure 2D). Notably, T0 expressed the highest levels of CCR8 among all Treg subsets (Figure 2H). Based on its enrichment in tumor tissue and in patients with advanced disease, its pronounced suppressive phenotype, and its high CCR8 expression relative to the other subsets, we defined T0 as the CCR8⁺ Treg state.

To place this state within Treg differentiation, we reconstructed pseudotime trajectories with Monocle 2. Tregs exhibited a developmental continuum from a root characterized by a naive phenotype to terminal states. Cells from tumors at early stages were concentrated near the origin, whereas those from tumors at advanced stages were enriched along the terminal branches, supporting the validity of our pseudotime analysis (Figure 2G). Branch expression analysis modeling (BEAM) analysis resolved a bifurcation from the progenitor pool into two terminal fates governed by four coordinated gene modules (Figure 2I): a classic effector and suppressive fate (CCR8⁺ Treg state) enriched for glycolysis and antigen processing programs, and a fate associated with stress adaptation (fate 2) marked by TNF signaling, efferocytosis, and xenobiotic metabolism. Representative dynamic genes reflected this divergence: CCR8⁺ Treg state retained functional markers such as GNLY and S100A4, whereas fate 2 showed increased expression of genes involved in stress responses and lipid metabolism, including HSPA1A, HSPA1B, and APOA2 (Figure 2J). These analyses identify CCR8⁺ Tregs as a terminally differentiated, hypersuppressive Treg state in HCC. To directly test the functional requirement for CCR8, we performed Treg-CD8⁺ T cell coculture suppression assays. Pharmacologic blockade of CCR8 significantly restored CD8⁺ T cell proliferation, increasing both the percent divided and the division index (Figure 2K), and enhanced IFN-γ production (Figure 2L). Thus, beyond the computational characterization, this experiment provides direct functional evidence that CCR8 signaling is required for the full suppressive activity of Tregs.

### Radiotherapy expands CCR8⁺ Tregs and reinforces their suppressive phenotype

We next examined how radiotherapy shapes intratumoral Tregs in two independent models: an intrahepatic orthotopic Hepa1-6 model (Figure 3A) and a subcutaneous Hepa1-6 model (Figure S2A). In the orthotopic model, both conventional radiotherapy (RT) and hypofractionated radiotherapy (HFRT) markedly increased intratumoral Treg infiltration and coordinately upregulated CTLA4 and CCR8 (Figure 3B-C), and the same pattern was reproduced in the subcutaneous model (Figure S2C, E, F), where HFRT also increased CD8⁺ T cell IFN-γ production (Figure S2D). HFRT produced the strongest phenotypic shift, with the highest fractions of FOXP3⁺CCR8⁺ and FOXP3⁺CTLA4⁺ Tregs. Consistent with these findings, paired pre- and post-radiotherapy human HCC specimens showed increased accumulation of CCR8⁺ Tregs after treatment, as assessed by multiplex immunofluorescence (Figure 3D, F).

To determine whether irradiation induces CCR8 expression in Tregs, we established a coculture system of Tregs and Hepa1-6 cells and exposed the coculture to different doses of irradiation (Figure 3E). CCR8 induction depended on the radiation dose and peaked at 4 Gy, with statistically significant induction at 2 Gy and 4 Gy relative to unirradiated controls (Figure 3G). Functionally, Tregs conditioned by radiation exerted stronger suppression of CD8⁺ T cell effector function and proliferation than control Tregs, as indicated by lower IFN-γ MFI (Figure 3H), a lower percentage of divided cells, and a reduced division index among cocultured CD8⁺ T cells labeled with CFSE (Figure 3I). Together, these findings indicate that radiotherapy not only increases Treg abundance but also intrinsically enhances the immunosuppressive capacity of Tregs in association with increased CCR8 expression.

### Radiotherapy promotes a CCR8⁺ Treg state with radioresistant and immunosuppressive programs

To characterize the cellular basis of this Treg state at high resolution, we generated an in-house murine single-cell atlas spanning normal liver, irradiated tumors, and anti-CCR8-treated tumors (Figure 4A; Figure S3A-C). Unsupervised subclustering of Tregs identified four subsets (T0-T3), among which cluster T2 was distinguished by a prominent CCR8⁺ Treg signature (Figure 4B-D). Pseudotime analysis revealed a bifurcating developmental trajectory originating from a Treg population with a naive phenotype. One branch (fate 2) progressed toward a terminal state with high CCR8 expression, whereas the other (fate 1) retained a transcriptional program resembling the baseline state (Figure 4E; confirmed with Monocle 3, Figure S3I). Along the branch characterized by high CCR8 expression, CCR8 levels increased sharply toward the terminal stage and were accompanied by progressive enrichment of the radioresistance signature (Figure 4F). Based on signature scoring and pseudotime analysis, we defined the T2 cluster as the CCR8⁺ Treg state. To characterize this population functionally, gene set enrichment analysis showed that the CCR8⁺ Tregs were enriched for multilayered DNA damage response and repair programs, including DDR, nonhomologous end joining, homologous recombination, and base-excision/single-strand break repair (Table S4), consistent with an enhanced capacity to tolerate cellular stress induced by radiation (Figure 4G). In parallel, CellChat analysis identified CCR8⁺ Tregs as a more prominent communication hub than CCR8⁻ Tregs within the irradiated TME, with higher outgoing and incoming interaction strength and preferential use of the MIF-(CD74/CD44) and ALCAM-CD6 axes to interact with myeloid cells and CD8⁺ T cells (Figure 4H-J; Figure S3J). To determine whether human and murine CCR8⁺ Treg states share the same biological program, we compared them using ortholog mapping, analysis of conserved signatures, and MetaNeighbor (Figure S3D-H). Human T0 and murine T2 showed the strongest correspondence (AUROC = 0.71), and the CCR8⁺ signature from each species showed the highest enrichment in the CCR8⁺ cluster of the other species, supporting conservation of the terminal CCR8⁺ Treg state. Together, these findings indicate that radiotherapy drives Tregs toward a CCR8⁺ state characterized by enhanced suppressive function, resistance to radiation, and extensive immunoregulatory interactions, and that this state is highly conserved between humans and mice.

### Spatial segregation of CCR8⁺ Tregs at the tumor periphery enforces a barrier against cytotoxic T cell infiltration

To define the spatial context of CCR8⁺ Tregs in HCC, we analyzed Visium spatial transcriptomic data from two independent tumors (CHC20 and CHC23; GSE245908) 22. Using robust cell type decomposition (RCTD) 23 together with histology, spots were assigned to four microanatomical niches: tumor core, myeloid-enriched, fibrotic stroma, and nontumor liver (Figure 5A). In both tumors, CCR8⁺ Tregs were preferentially enriched in peritumoral myeloid-enriched and fibrotic regions, whereas the tumor core was largely devoid of these cells (Figure 5B). CD8⁺ T cells were also sparse in the tumor core, consistent with a phenotype characterized by immune exclusion 24. Spatial distance analysis revealed a consistent decrease in CD8⁺ T cell representation toward the tumor core in both samples. In CHC23, spots enriched in CCR8⁺ Tregs predominated within 300 μm of the tumor boundary, whereas spots dominated by CD8⁺ T cells became more prevalent at intermediate distances. Similarly, in CHC20, no clear predominance of CD8⁺ T cell spots was observed within 700 μm, and their relative abundance increased only at greater distances (Figure 5C). Spatial analysis of ligand and receptor interactions further identified barrier-organizer axes from myeloid and fibroblast senders targeting CCR8⁺ Tregs, and barrier-associated axes from myeloid, CCR8⁺ Treg, and fibroblast senders targeting CD8⁺ T cells (Figure S4C-D). These findings support a model in which CCR8⁺ Tregs form a suppressive peritumoral shell that spatially limits cytotoxic T cell access to tumor nests.

Multiplex immunofluorescence of four paired pre- and post-radiotherapy HCC specimens provided orthogonal support for this architecture (Figure 5E). Although the overall COL1A1⁺ stromal area remained largely unchanged after radiotherapy, the density of CCR8⁺ Tregs within fibrotic stroma increased significantly, and the CCR8⁺/CD8⁺ ratio rose further, indicating a shift toward a more suppressive stromal immune contexture after treatment (Figure 5D). Cell-cell communication analyses of the preceding murine scRNA-seq data and spatial transcriptomic data from the two human tumor specimens indicated strong intercellular communication between CCR8⁺ Tregs and myeloid cells, with M2-like TAMs emerging as the predominant cellular source of CCL1 15,18. These findings prompted us to investigate whether the CCL1-CCR8 axis drives CCR8⁺ Treg recruitment and whether this axis is modulated by radiotherapy. In Transwell assays, both recombinant CCL1 and M2-like TAM-conditioned medium significantly enhanced migration of Tregs compared with basal controls, and TAM-conditioned medium-induced migration was attenuated by anti-CCR8, confirming CCR8 dependence (Figure 5F-G). Importantly, ELISA showed that irradiation (4 Gy) significantly increased CCL1 secretion by M2-like TAMs, whereas CCL1 secretion by Hepa1-6 tumor cells was unchanged (Figure 5H). Thus, radiotherapy actively strengthens the CCL1-CCR8 recruitment signal, which originates from CCL1 produced by myeloid cells and acts through CCR8 on Tregs, rather than merely expanding CCR8⁺ Tregs *in situ*.

### Hypofractionated radiotherapy combined with CCR8 blockade remodels the immunosuppressive microenvironment and improves tumor control

To determine whether anti-CCR8 could improve the response to HFRT, mice bearing Hepa1-6 tumors received control treatment, anti-CCR8, HFRT, or the combination in two complementary models: an intrahepatic orthotopic Hepa1-6-luc model monitored by bioluminescence imaging and a subcutaneous Hepa1-6 model monitored by caliper measurement (Figure S2B). In the orthotopic model, bioluminescence total flux showed that the combination achieved the most pronounced suppression of tumor progression throughout the observation period relative to either monotherapy (Figure 6A-B, D). Concordantly, in the subcutaneous model, tumor growth curves, endpoint tumor weight, and cumulative tumor burden (area under the growth curve) were all lowest in the combination group. These findings showed greater tumor control with HFRT plus anti-CCR8 than with either monotherapy in these models (Figure 6C, E-G). Body weight did not differ among groups, indicating that the treatments were well tolerated and that the greater tumor control was not accompanied by apparent systemic toxicity or loss of animal condition. The liver/body-weight ratio, which in this model reflects intrahepatic tumor burden, was lowest in the combination group, consistent with the bioluminescence data (Figure 6H-I).

We next asked whether improved tumor control was accompanied by remodeling of the suppressive Treg compartment. In the orthotopic model, flow cytometry showed that HFRT alone increased the proportion of FOXP3⁺CCR8⁺ and FOXP3⁺CTLA4⁺ Tregs, whereas adding anti-CCR8 to HFRT significantly reduced these suppressive Treg populations relative to HFRT alone (Figure 6J, L), with concordant changes in the subcutaneous model (Figure S2G-I). Because effective antitumor immunity ultimately depends on cytotoxic T cell function, we evaluated CD8⁺ T cell activation. IFN-γ intensity was highest in the combination group (Figure 6K, M; Figure S2J). Collectively, the combination of HFRT and anti-CCR8 achieved greater tumor control than either treatment alone, while reducing the accumulation of suppressive CCR8⁺ and CTLA4⁺ Tregs following RT and enhancing CD8⁺ T cell effector function.

### Radiation activates c-Rel to promote CCR8 transcription and reinforce the suppressive Treg program

Having shown that radiotherapy expands CCR8⁺ Tregs and that CCR8 blockade improves tumor control, we next investigated how radiation induces CCR8 expression in Tregs. Pathway enrichment analysis comparing irradiated and control conditions identified TNFA signaling via NF-κB as the most strongly upregulated program following radiation (Figure 7A). Consistent with this finding, immunoblotting showed that radiation increased phosphorylation of NF-κB p65 in Tregs, whereas total p65 remained unchanged (Figure 7B). These results identified activation of NF-κB as a potential link between radiation and CCR8 induction.

To identify the transcriptional regulator associated with the CCR8⁺ Treg state, we applied SCENIC to Treg subsets from the GSE149614 dataset. REL, which encodes c-Rel, a member of the NF-κB family 25,26, showed preferential regulon activity in CCR8⁺ Tregs (Figure 7C). We then examined whether REL contributes to the stability of this cell state using an in silico knockout analysis based on the gene regulatory network. Simulated REL deletion destabilized the T0 state and redirected the perturbed cells predominantly toward the transcriptional program of T1 (Figure 7D-E). Together, these computational analyses identified REL as a candidate regulator of the terminal CCR8⁺ Treg state.

To determine whether c-Rel directly controls CCR8 transcription, we examined the CCR8 promoter and identified putative c-Rel-binding sites (Figure 7G). ChIP using an antibody against c-Rel showed significant enrichment of the CCR8 promoter relative to the IgG control, demonstrating promoter occupancy by c-Rel (Figure 7H). In reporter assays, REL overexpression increased the activity of the unmutated CCR8 promoter. Substitution of Mut1 reduced reporter activity to the level observed with the empty vector, whereas Mut2 and Mut3 retained their responses to REL overexpression (Figure 7I-J). Together, the ChIP and reporter results demonstrated that c-Rel directly binds to and activates the CCR8 promoter, with Mut1 providing the clearest evidence of sequence dependence.

Finally, we tested whether c-Rel is required for the increase in CCR8 expression and Treg suppressive function induced by radiation. After confirming efficient REL knockdown with si-Rel (Figure 7F), flow cytometry showed that radiation increased the proportions of p-c-Rel⁺ and CCR8⁺ cells among FOXP3⁺ Tregs. Both increases were prevented by REL knockdown (Figure 7K). Functionally, Tregs conditioned by radiation reduced IFN-γ production and proliferation in cocultured CD8⁺ T cells. REL knockdown restored IFN-γ expression, the division index, and the percentage of divided CD8⁺ T cells (Figure 7L-O). Together, these results show that radiation activates c-Rel, which directly promotes CCR8 transcription and reinforces the suppressive CCR8⁺ Treg program.

## Discussion

In this study, we show that radiation activates NF-κB signaling and that c-Rel directly promotes CCR8 transcription, thereby reinforcing a highly suppressive Treg state associated with spatial immune restriction, disease progression, and poor radiotherapy outcomes in HCC. A distinguishing feature of this work is the integration of public bulk, single-cell, and spatial transcriptomic cohorts with newly generated experimental data, including an in-house murine single-cell atlas, paired human specimens collected before and after radiotherapy, *in vivo* combination therapy with bioluminescence monitoring, and a series of functional and molecular assays. Rather than treating CCR8 simply as a marker of Treg abundance 27, our data position CCR8 within a functional program that links suppressive specialization, spatial immune exclusion, and radiotherapy response, while providing direct evidence that c-Rel binds to and activates the CCR8 promoter.

A central implication is that Treg heterogeneity within HCC tumors is better understood through specialization into distinct functional states rather than through Treg markers alone. Our analyses support a CCR8⁺ Treg program characterized by enhanced suppressive features and the progressive acquisition of traits associated with resistance to radiation. Comparisons between species further showed that this program is conserved in human and murine tumors. These findings suggest that the HCC microenvironment does not uniformly expand all Tregs but instead favors a functionally reinforced subset with a greater capacity to persist under treatment pressure. In this context, selective targeting of CCR8⁺ Tregs may be more relevant than broad Treg depletion. This is where our study extends prior work: strategies targeting broadly expressed Treg markers such as CD25 are limited by poor selectivity and systemic toxicity 13, whereas earlier CCR8 studies focused mainly on depletion with antibodies in combination with immune checkpoint blockade 14,16. Our findings indicate that radiotherapy expands and reinforces a transcriptionally defined CCR8⁺ Treg state and that combining CCR8 blockade with radiotherapy remodels the suppressive immune niche.

Our spatial analyses place this program within a defined tissue context. In both spatial samples, CCR8⁺ Tregs were enriched in peritumoral myeloid- and fibrosis-associated regions, whereas CD8⁺ T cells showed a distribution consistent with immune exclusion 24. Migration assays showed that CCL1 recruits Tregs through CCR8 and that radiation directly increases CCL1 production by M2 macrophages but not by tumor cells. Together, these results support a model in which CCL1 and CCR8 signaling establishes a peritumoral immunoregulatory barrier that limits cytotoxic T cell access to malignant hepatocyte nests.

Beyond CCR8, our consensus feature selection also nominated TNFRSF4 (OX40), TNFRSF9 (4-1BB), and RTKN2. These genes are biologically plausible contributors to the suppressive phenotype. TNFRSF4 and TNFRSF9 are costimulatory molecules belonging to the TNF receptor superfamily that are highly expressed by activated Tregs within tumors and are associated with their expansion and stability 28. RTKN2 is a relatively understudied Treg signature gene that may contribute to lymphocyte survival 29. Although we focused mechanistically on CCR8, these candidates warrant dedicated functional study and may cooperate with CCR8 in sustaining the terminal suppressive state.

Mechanistically, our data indicate that radiotherapy amplifies a suppressive circuit in the TME rather than simply overcoming it. Radiation activated NF-κB signaling, while the REL regulon showed preferential activity in CCR8⁺ Tregs and c-Rel directly occupied and activated the CCR8 promoter. Consistent with this mechanism, REL knockdown prevented the increase in CCR8 following radiation and restored IFN-γ production and proliferation in cocultured CD8⁺ T cells. These findings link inflammatory signaling induced by radiation to the reinforcement of a suppressive Treg program. They may help explain why radiotherapy can promote effector immune activity while also eliciting a compensatory regulatory response. The enrichment of DNA repair programs further characterized the CCR8⁺ Treg state as adapted to stress related to treatment, although these analyses did not establish that c-Rel directly controls the repair program. Therapeutically, CCR8 blockade enhanced the efficacy of HFRT, reduced the suppressive Treg population induced by radiation, and increased CD8⁺ T cell activity. Across the tested models, the combination achieved greater tumor control than either treatment alone, supporting CCR8 as a selective target for combining locoregional radiotherapy with immune modulation.

Several limitations should be acknowledged. First, although we used both intrahepatic and subcutaneous models, additional work in spontaneous liver tumor models will be important to refine the spatial and immunological relevance of the CCR8 axis. Second, the paired human pre- and post-radiotherapy cohort was small, so the human trends, while concordant with the overall model, require validation in larger, clinically annotated cohorts before strong translational conclusions can be drawn. Third, some spatial analyses are descriptive, and the combination experiments were not designed to establish formal pharmacologic synergy. Larger studies with prespecified interaction analyses are needed. Nevertheless, the consistency across independent human cohorts, cross-species single-cell analyses, radiotherapy-treated human specimens, and direct molecular perturbation supports the robustness of the central model.

In summary, our findings support a model in which radiotherapy induces adaptive immune resistance in HCC through activation of NF-κB signaling and direct transcriptional regulation of CCR8 by c-Rel. This response expands and reinforces a highly suppressive CCR8⁺ Treg program localized at the peritumoral interface. By integrating Treg state specialization, spatial immune exclusion, treatment response, and direct experimental evidence that c-Rel regulates CCR8 transcription, this study provides a rationale for targeting CCR8 to improve the durability of responses to radiotherapy in HCC.

## Supplementary Material

Supplementary materials and methods, figures and tables.

## Figures and Tables

**Figure 1:**
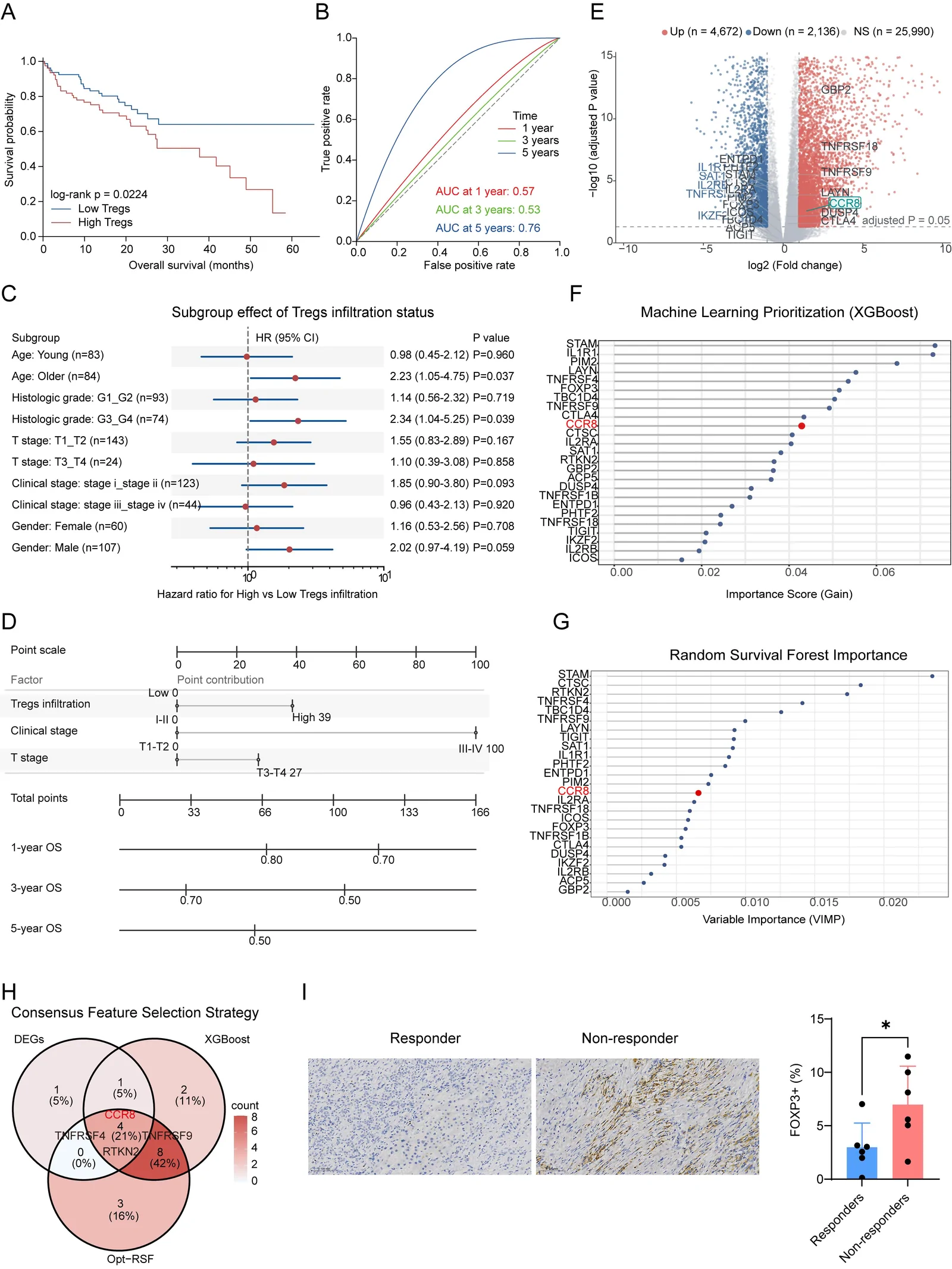
** TCGA-LIHC analysis links high Treg infiltration to poor prognosis and identifies CCR8 as a key candidate.** (A) Kaplan-Meier overall survival (OS) analysis comparing Treg-high versus Treg-low patients. (B) Time-dependent ROC curves evaluating the predictive performance of Treg abundance for 1-, 3-, and 5-year OS. (C) Subgroup forest plot showing hazard ratios (HRs) for Treg infiltration status across clinical strata; bars denote 95% confidence intervals. (D) Nomogram integrating Treg infiltration with clinical stage and T stage to estimate 1-, 3-, and 5-year OS. (E) Volcano plot of DEGs between tumor and normal tissues in TCGA-LIHC. (F) XGBoost feature-importance ranking (gain). (G) Random survival forest permutation variable importance (VIMP). (H) Intersection of DEGs with features selected by XGBoost and RSF, identifying four candidates. (I) Representative FOXP3 immunohistochemistry in responders versus nonresponders and quantification of FOXP3⁺ cells (n = 6 patients/group). Survival differences were assessed by log-rank test; HRs were estimated using Cox proportional hazards models. Differences in FOXP3⁺ cell frequency between responders and nonresponders were assessed by two-tailed Student's t-test (*P < 0.05).

**Figure 2:**
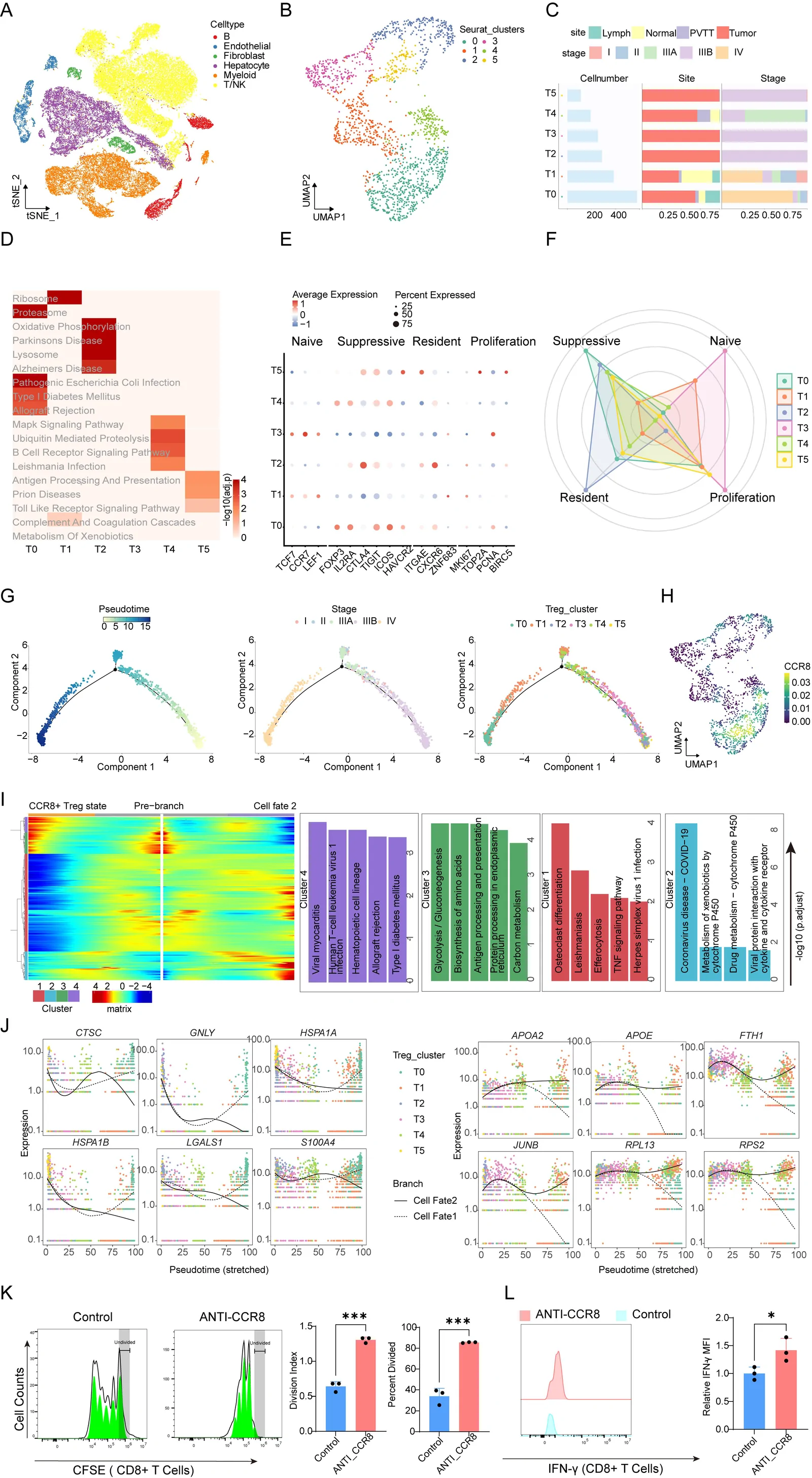
** Analysis of single cells identifies a CCR8⁺ Treg state enriched in advanced disease and shows that CCR8 blockade reduces Treg suppression.** (A) t-SNE overview of major cell populations in the GSE149614 HCC scRNA-seq dataset. (B) UMAP of FOXP3⁺ Tregs showing unsupervised clustering into six subsets (T0-T5). (C) Distribution of Treg clusters by cell number, tissue site, and clinical stage. (D) KEGG pathway signatures enriched in each Treg cluster. (E) Dot plot of signature markers (naive, suppressive, resident, proliferation) across Treg clusters. (F) Radar plot comparing the four phenotype dimensions across clusters. (G) Monocle 2 trajectory reconstruction of Tregs, colored by pseudotime, clinical stage, and cluster. (H) Feature plot of CCR8 expression across the Treg UMAP. (I) BEAM gene-module heatmap along pseudotime with KEGG enrichment for each of the four gene modules. (J) Representative genes with dynamic expression along pseudotime in the two fate branches. (K) Representative CFSE dilution profiles and quantification of division index and percent divided of CD8⁺ T cells in Treg-CD8 coculture under control versus anti-CCR8 conditions (n = 3). (L) Representative IFN-γ histograms and quantification of relative IFN-γ MFI in CD8⁺ T cells (n = 3). P values by two-tailed Student's t-test (***P < 0.001, **P < 0.01, *P < 0.05).

**Figure 3:**
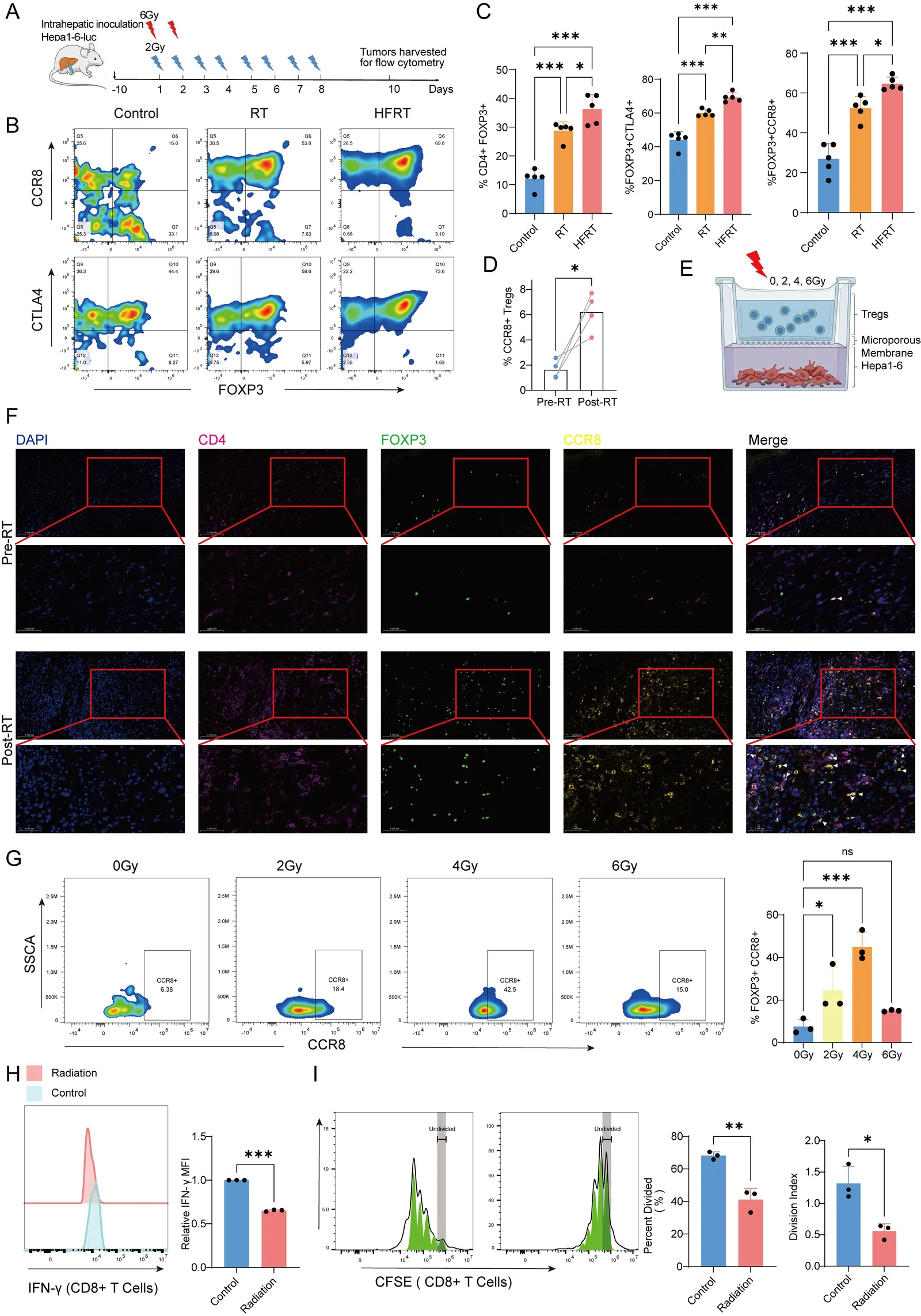
** Radiotherapy reprograms Tregs toward a CCR8⁺ state with potentiated immunosuppressive capacity.** (A) Schematic of the intrahepatic Hepa1-6 model. (B) Representative flow cytometry plots of intratumoral CCR8 and CTLA4 versus FOXP3 under control, RT, and HFRT. (C) Quantification of %CD4⁺FOXP3⁺, %FOXP3⁺CTLA4⁺, and %FOXP3⁺CCR8⁺ cells (n = 5 mice/group). (D) Paired quantification of %CCR8⁺ Tregs in pre-RT versus post-RT human tumor samples (n = 4 patients). (E) Schematic of the Transwell coculture system. (F) Representative multiplex immunofluorescence of paired human HCC before and after radiotherapy (DAPI, CD4, FOXP3, CCR8, merge). (G) Representative flow cytometry plots and quantification of CCR8 induction in Tregs after irradiation of the Treg-Hepa1-6 coculture at 0, 2, 4, and 6 Gy (n = 3). (H) Representative IFN-γ histograms and quantification of relative IFN-γ MFI in CD8⁺ T cells after coculture with control versus radiation-conditioned Tregs (n = 3). (I) Representative CFSE dilution profiles and quantification of percent divided and division index of CD8⁺ T cells (n = 3). P values by one-way ANOVA (C, G), two-tailed Student's t-test (H, I), and paired t-test (D) (***P < 0.001, **P < 0.01, *P < 0.05; ns, not significant).

**Figure 4:**
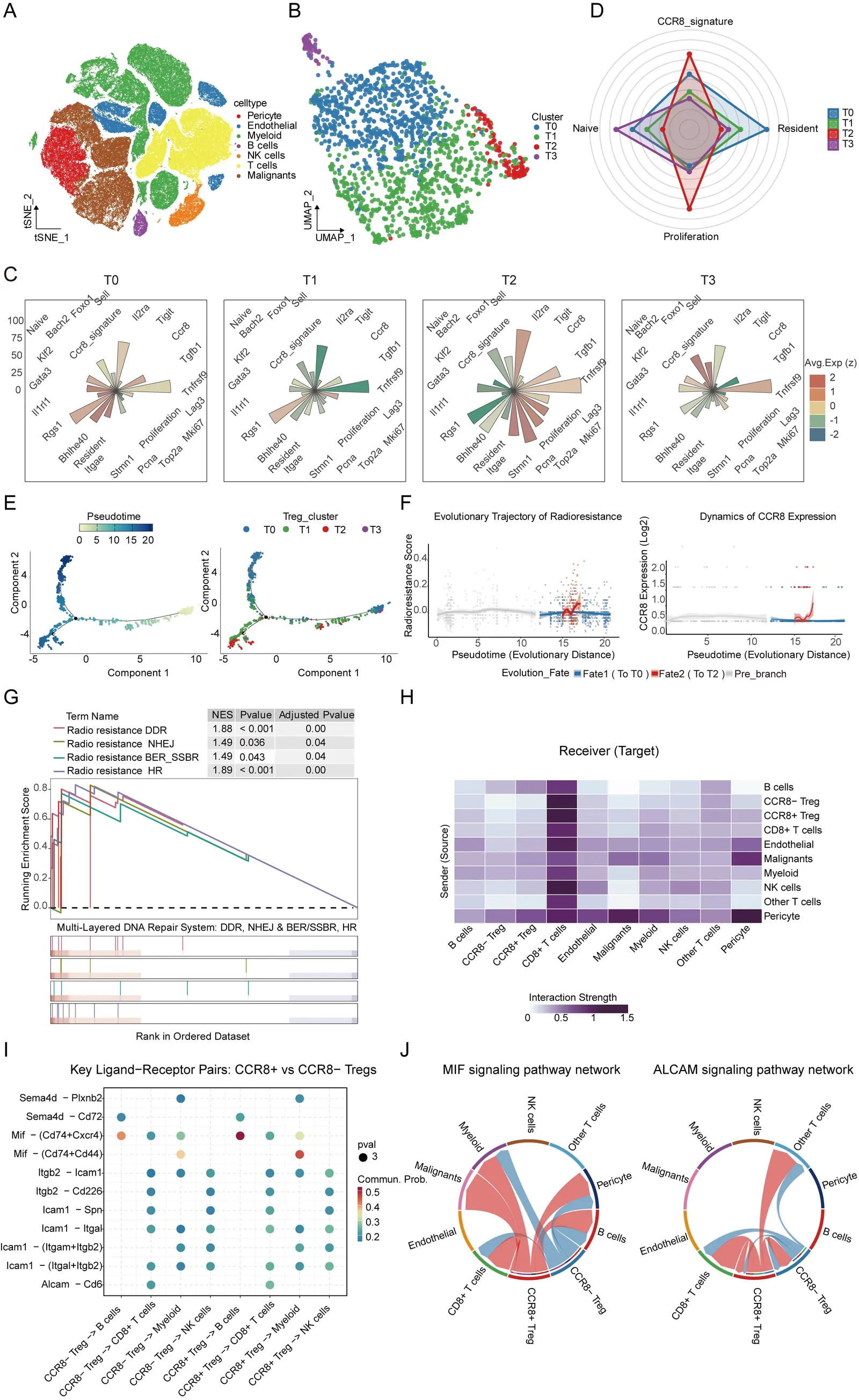
** Analysis of single cells from our internally generated dataset reveals that radiotherapy drives murine Tregs toward a terminal CCR8⁺ state with radioresistant and immunosuppressive features.** (A) t-SNE map of the integrated murine HCC single-cell atlas. (B) UMAP of the Treg compartment subclustered into four subsets (T0-T3). (C) Rose plots of marker expression (z-score) per Treg subset. (D) Radar plot of functional-state scores (CCR8 signature, resident, proliferation, naive) across subsets. (E) Monocle 2 trajectory colored by pseudotime and Treg cluster. (F) Dynamics of the radioresistance score and CCR8 expression along the evolutionary trajectory. (G) GSEA of multilayered DNA-repair/radioresistance programs (DDR, NHEJ, BER/SSBR, HR) with NES, P values, and adjusted P values. (H) CellChat sender-receiver interaction-strength heatmap. (I) Dot plot of prioritized ligand-receptor pairs comparing CCR8⁺ and CCR8⁻ Treg-centered communication. (J) Network visualizations of the MIF and ALCAM signaling pathways centered on CCR8⁺ Tregs.

**Figure 5:**
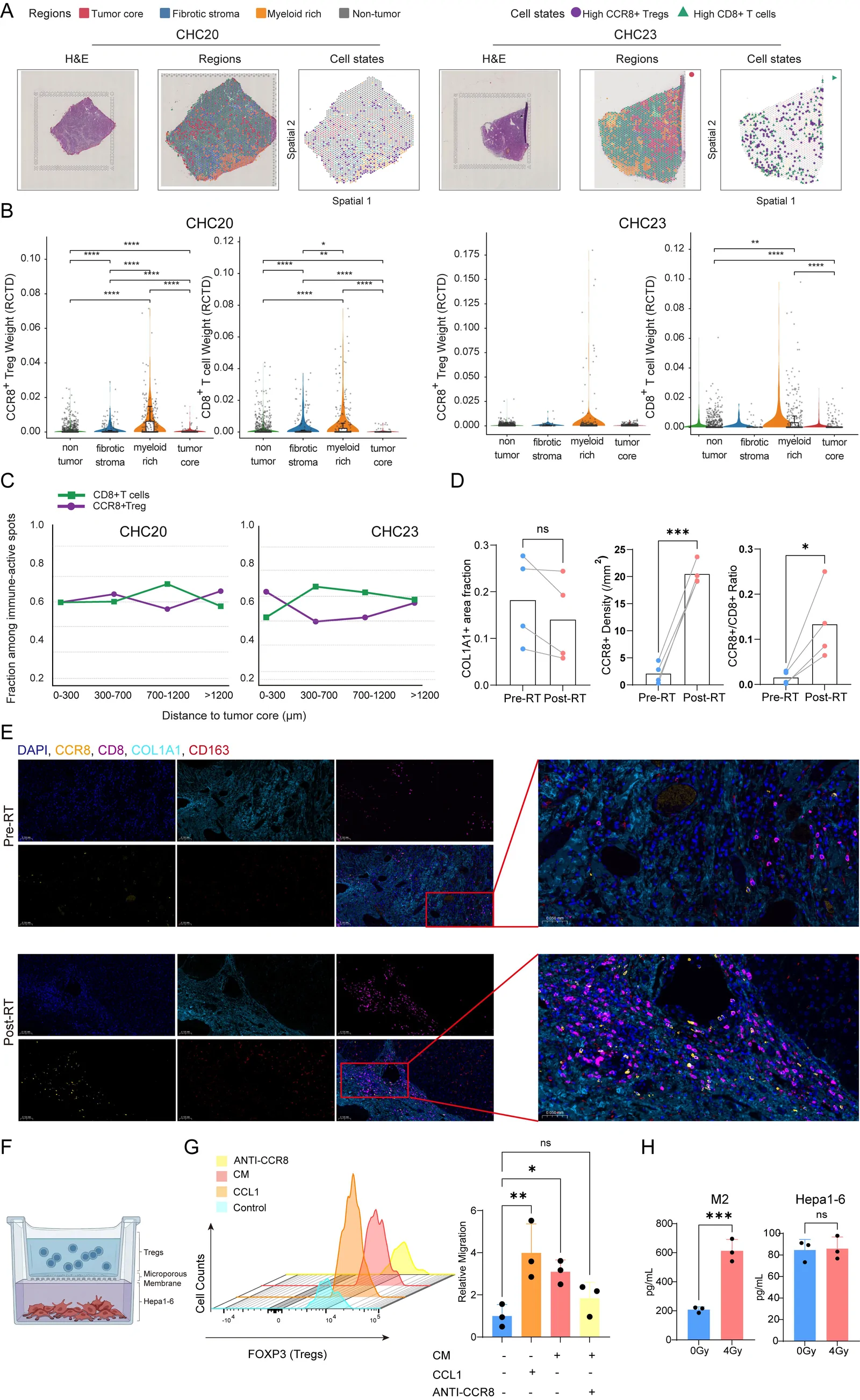
** Spatial transcriptomics revealed the enrichment of CCR8⁺ Tregs within peritumoral myeloid and fibrotic niches and identified recruitment enhanced by radiation and mediated by CCL1.** (A) Spatial analysis of CHC20 and CHC23 guided by histology, showing H&E staining, niche annotation using RCTD, and spots enriched in CCR8⁺ Tregs or CD8⁺ T cells. (B) RCTD-inferred CCR8⁺ Treg and CD8⁺ T cell weights across the four niches in each sample. (C) Fraction of spots with immune activity at increasing distances from the tumor core in CHC20 and CHC23. (D) Quantification of multiplex immunofluorescence within fibrotic stroma from four paired pre-RT and post-RT specimens (n = 4 patients). (E) Representative multiplex immunofluorescence of paired pre-RT and post-RT HCC tissues (DAPI, CCR8, CD8, COL1A1, CD163). (F) Schematic of the Transwell migration assay. (G) Representative migration profiles and quantification of relative migration of Tregs toward basal medium (control), recombinant CCL1, M2-like TAM-conditioned medium (CM), or CM plus anti-CCR8 (n = 3). (H) ELISA quantification of secreted CCL1 from M2-like TAMs and from Hepa1-6 cells irradiated at 0 versus 4 Gy (n = 3). P values by one-way ANOVA (G), Kruskal-Wallis test with two-sided Mann-Whitney U post hoc tests and Benjamini-Hochberg correction (B), two-tailed Student's t-test (H), or paired t-test (D) (****P < 0.0001, ***P < 0.001, **P < 0.01, *P < 0.05; ns, not significant).

**Figure 6:**
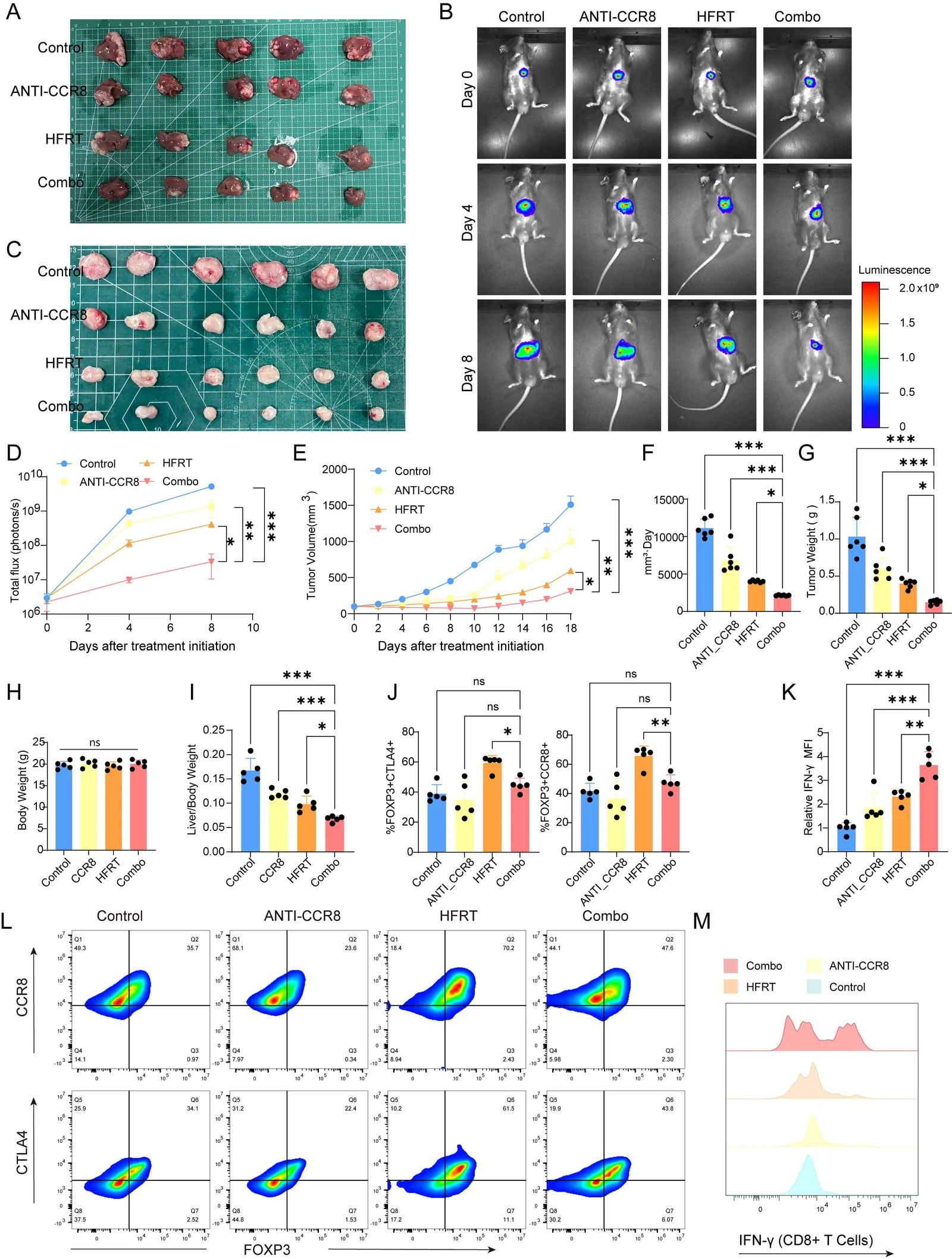
** Hypofractionated radiotherapy combined with anti-CCR8 reduces CCR8⁺ suppressive Tregs and enhances CD8⁺ T cell effector function.** (A) Representative gross images of excised livers bearing intrahepatic tumors at endpoint (n = 5 mice/group). (B) Representative bioluminescence images at Day 0, 4, and 8. (C) Representative images of excised subcutaneous tumors at endpoint (n = 6 mice/group). (D) Bioluminescence total flux over time (n = 5 mice/group). (E) Tumor growth curves (n = 6 mice/group). (F) Cumulative tumor burden (n = 6 mice/group). (G) Terminal tumor weight (n = 6 mice/group). (H) Body weight (n = 5 mice/group). (I) Liver/body-weight ratio (n = 5 mice/group). (J) Quantification of %FOXP3⁺CTLA4⁺ and %FOXP3⁺CCR8⁺ Tregs (n = 5 mice/group). (K) Relative IFN-γ MFI in CD8⁺ T cells (n = 5 mice/group). (L) Representative flow cytometry plots of CCR8 and CTLA4 versus FOXP3 in the four groups. (M) Representative IFN-γ histograms in CD8⁺ T cells. Panels A, B, D, and H-M are from the intrahepatic orthotopic model and panels C and E-G from the subcutaneous model. P values by one-way ANOVA (F-K) or two-way repeated-measures ANOVA (D, E) (***P < 0.001, **P < 0.01, *P < 0.05; ns, not significant).

**Figure 7:**
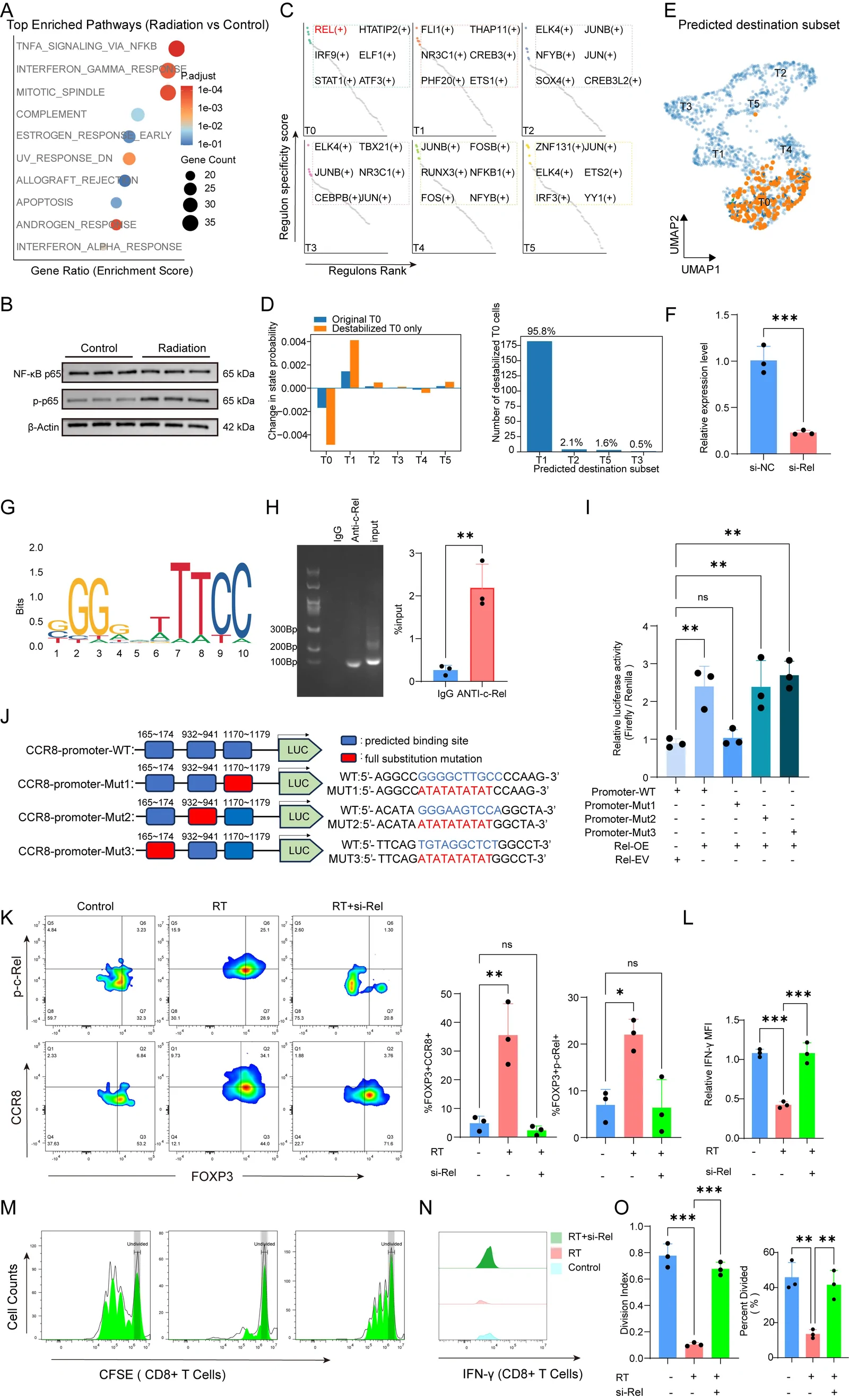
** Radiation activates NF-κB signaling dependent on REL to directly transactivate CCR8 and establish the suppressive CCR8⁺ Treg program.** (A) Pathway-enrichment analysis of irradiated versus control conditions. (B) Immunoblot of NF-κB p65, p-p65, and β-actin in Tregs. (C) SCENIC regulon-specificity ranking across Treg subsets. (D) Probability-space state redistribution following REL in silico knockout. (E) REL in silico knockout projected onto the integrated UMAP. Pale blue dots, all Tregs (T0-T5); orange dots, destabilized T0 cells; arrows, predicted displacement, colored by destination state (blue, T1; orange, T2; green, T5). (F) siRNA knockdown efficiency of c-Rel (REL) in Tregs (n = 3). (G) Predicted REL-binding motif at the CCR8 promoter. (H) ChIP of c-Rel at the CCR8 promoter: representative agarose gel of ChIP-PCR products (IgG, anti-c-Rel, input) and ChIP-qPCR quantification expressed as % input (n = 3). (I) CCR8 promoter luciferase reporter activity for wild-type and binding-site mutant constructs with REL overexpression (Rel-OE) or empty vector (Rel-EV) (n = 3). (J) Schematic of the CCR8 promoter binding sites and full-substitution mutations. (K) Representative flow cytometry plots and quantification of p-c-Rel and CCR8 in FOXP3⁺ Tregs under control, RT, and RT plus si-Rel (n = 3). (L) Relative IFN-γ MFI in cocultured CD8⁺ T cells (n = 3). (M) Representative CFSE dilution histograms. (N) Representative CD8⁺ T cell IFN-γ histograms. (O) Quantification of division index and percent divided of CD8⁺ T cells (n = 3). P values by one-way ANOVA (I, K, L, O) or two-tailed Student's t-test (F, H) (***P < 0.001, **P < 0.01, *P < 0.05; ns, not significant).

## Data Availability

The Methods section and Supplementary Materials and Methods describe all data used in this study. The data and code supporting the conclusions of this work will be provided by the corresponding author on reasonable request.

## Abbreviations

AUC: area under the curve
AUROC: area under the receiver operating characteristic curve
BEAM: branch expression analysis modeling
BER: base-excision repair
BMDM: bone marrow-derived macrophage
CCR8: C-C chemokine receptor 8
CFSE: carboxyfluorescein succinimidyl ester
ChIP: chromatin immunoprecipitation
DDR: DNA damage response and repair
DEGs: differentially expressed genes
ELISA: enzyme-linked immunosorbent assay
GEO: Gene Expression Omnibus
GSEA: gene set enrichment analysis
GSVA: gene set variation analysis
HCC: hepatocellular carcinoma
HFRT: hypofractionated radiotherapy
HR: hazard ratio (survival analyses) or homologous recombination (DNA repair analyses)
ICGC: International Cancer Genome Consortium
IFN-γ: interferon-γ
IHC: immunohistochemistry
KEGG: Kyoto Encyclopedia of Genes and Genomes
LIHC: liver hepatocellular carcinoma
MFI: mean fluorescence intensity
mIF: multiplex immunofluorescence
MIF: macrophage migration inhibitory factor
mRECIST: modified Response Evaluation Criteria in Solid Tumors
NES: normalized enrichment score
NF-κB: nuclear factor kappa-light-chain-enhancer of activated B cells
NHEJ: nonhomologous end joining
OS: overall survival
RCTD: robust cell-type decomposition
ROC: receiver operating characteristic
RSF: random survival forest
RT: radiotherapy
SCENIC: single-cell regulatory network inference and clustering
scRNA-seq: single-cell RNA sequencing
SSBR: single-strand break repair
TAM: tumor-associated macrophage
TCGA: The Cancer Genome Atlas
TME: tumor microenvironment
Tregs: regulatory T cells
t-SNE: t-distributed stochastic neighbor embedding
UMAP: uniform manifold approximation and projection
VIMP: variable importance
