# Supplementary material for: Radiotherapy Reprograms CCR8⁺ Regulatory T Cells via REL-NF-κB to Enforce Spatial Immune Exclusion in Hepatocellular Carcinoma
Source: Int J Biol Sci. 2026 Sep 24;22(15):8476–91. doi: 10.7150/ijbs.136364 (PMC13685768; doi:10.7150/ijbs.136364)
Supplement: Supplementary file 1 [file ijbsv22p8476s1.pdf]

Supplementary Information

Radiotherapy Reprograms CCR8^{+} Regulatory T Cells via REL–NF-κB to Enforce Spatial Immune Exclusion in Hepatocellular Carcinoma

Huanliang Chen, Shuxuan Wang, Bufu Tang, Yanyan Lin, Pan Zhou, Jiaoyang Yang, Qianqian Zhao, Zhaochong Zeng

Table of Contents

• Supplementary Materials and Methods

• Supplementary Figures (Figures S1–S4)

• Supplementary Tables (Tables S1–S4)

Supplementary Materials and Methods

Reference numbers cited in this section correspond to the reference list of the main manuscript.

TCGA and ICGC cohort analysis

RNA-seq transcriptomic profiles and corresponding clinical information of HCC patients were obtained from The Cancer Genome Atlas (TCGA-LIHC) and the International Cancer Genome Consortium (ICGC-LIHC) databases. All transcriptomic data were processed in R (version 4.2.1). Gene identifiers were converted to official gene symbols, and duplicated symbols were collapsed by the median expression value.

Treg infiltration levels were estimated using single-sample gene set enrichment analysis (ssGSEA) [30] based on a predefined Treg-related gene signature. The enrichment score of each sample was calculated from normalized transcriptomic profiles, and patients were stratified into Treg-high and Treg-low groups according to the median Treg score. Kaplan–Meier survival analysis with the log-rank test was performed to compare overall survival (OS) between groups. Time-dependent receiver operating

characteristic (ROC) curves were generated using the timeROC package to evaluate the predictive performance of the Treg score for 1-, 3-, and 5-year OS. Univariate and multivariate Cox proportional hazards regression analyses were performed to determine whether Treg infiltration was an independent prognostic factor after adjustment for the clinicopathological variables available in each cohort. A prognostic nomogram integrating the Treg score with the independent clinical predictors identified in each cohort was constructed using the rms package.

Identification of Treg-associated genes using machine learning

Differentially expressed genes (DEGs) between HCC tumor tissues and adjacent nontumor tissues were identified from TCGA-LIHC data using the limma package [31]; genes with $|\log2 \text{ fold change}| > 1$ and false discovery rate (FDR) < 0.05 were considered significant. To identify robust Treg-associated candidate genes, a multistep machine-learning framework was applied. Random survival forest analysis was performed with the randomForestSRC package (1,000 trees), and gene importance was evaluated by permutation importance (VIMP) [32]. In parallel, an XGBoost model [33] was constructed to assess feature importance based on gain values. Genes ranked among the top candidates by both machine-learning approaches were intersected with the DEG set to generate a core Treg-associated gene signature (CCR8, TNFRSF4, TNFRSF9, RTKN2).

Single-cell RNA sequencing data processing and analysis

Publicly available human HCC scRNA-seq data (GSE149614) and in-house murine scRNA-seq datasets were analyzed. Human data (expression matrix and clinical annotation) were obtained from GEO. Raw count matrices were processed using Seurat in R [34]. Cells with fewer than 500 or more than 8,000 detected genes, fewer than

1,000 total transcripts, mitochondrial transcript proportions exceeding 5%, or hemoglobin gene contamination exceeding 1% were excluded. Following quality control, data were normalized using LogNormalize, and the top 2,000 highly variable genes were selected for dimensionality reduction. Principal component analysis (PCA) was performed and the first 30 principal components were used for integration, clustering, and visualization. Batch effects among human samples were corrected using Seurat reciprocal PCA (RPCA)-based integration (FindIntegrationAnchors and IntegrateData). Integrated data were visualized by t-distributed stochastic neighbor embedding (t-SNE), and major cell populations were annotated by canonical lineage markers. For Treg-focused analysis, T/NK cells were extracted and reclustered; Tregs were identified by canonical markers (FOXP3, IL2RA, CTLA4, TNFRSF18), and CCR8-expressing Treg populations were characterized by subclustering, differential expression, and module scoring.

Murine scRNA-seq libraries were generated using the 10x Genomics Chromium platform across three independent batches (Figure S3A) according to the manufacturer's standard protocol and processed with Cell Ranger. Sequencing quality was evaluated using Cell Ranger metrics (barcode validity, UMI quality, sequencing saturation, and genome alignment), and all libraries retained for downstream analysis met these quality criteria. Cells with fewer than 200 or more than 6,000 detected genes, mitochondrial transcript proportions exceeding 15%, or low transcriptomic complexity were excluded. Potential doublets were identified and removed with DoubletFinder [35], with expected doublet numbers estimated from the total recovered cell number and adjusted for homotypic doublet proportions. Potential ambient-RNA artifacts were evaluated during preprocessing and minimized through stringent quality filtering and marker-based annotation. Across the 16 murine libraries, 13,096–14,975 high-quality cells were

retained after quality control and doublet removal, with a mean sequencing depth of 20,335–28,449 reads per cell and a median of 2,074–3,584 genes detected per cell. Murine data were normalized with LogNormalize, followed by variable-gene selection, scaling, PCA, and dimensionality reduction; batch effects among murine samples were corrected using Harmony [36] based on sample identity, and the first 30 principal components were used for neighborhood-graph construction, clustering, and t-SNE visualization of the global atlas. $CD4^+FOXP3^+$ Tregs were extracted for secondary analysis, reprocessed using SCTransform, PCA, Harmony integration, and UMAP clustering, and differential expression among Treg clusters was assessed with FindMarkers.

Cross-species conservation analysis

To test whether the human and murine $CCR8^+$ Treg states represent a conserved program, one-to-one orthologs between human and mouse were identified (14,505 ortholog pairs). A core conserved Treg signature was derived from shared differentially expressed genes, and its activity was scored in human and murine Treg clusters using module scoring. Transcriptional similarity between human (T0–T5) and murine (T0–T3) Treg clusters was quantified with MetaNeighbor [37] (AUROC), and reciprocal signature scoring (mouse T2 signature in human Tregs and human T0 signature in murine Tregs) was performed to confirm correspondence between the two $CCR8^+$ terminal states.

Pseudotime trajectory inference

Pseudotime analysis was performed with Monocle 2 [38] (and confirmed with Monocle 3 [39]). Treg subsets were converted into CellDataSet objects using raw expression matrices; differentially expressed genes among Treg clusters were selected as ordering

genes, followed by DDRTree dimensionality reduction and cell ordering. Naive-like Tregs were assigned as the developmental origin, and branch-dependent genes were identified using branch expression analysis modeling (BEAM). Dynamic genes along pseudotime were analyzed to characterize transitions associated with suppressive activity, stress adaptation, and radiotherapy-associated states.

SCENIC analysis and transcription factor regulon activity

Gene regulatory networks were inferred using the SCENIC pipeline [40] on normalized scRNA-seq matrices. Transcription factor–target coexpression modules were identified with GRNBoost2; candidate regulons were refined by motif-enrichment analysis with RcisTarget; and regulon activity in each cell was quantified with AUCell. To identify regulators that are specifically active in a given Treg state, we computed the regulon-specificity score (RSS) for each regulon within the SCENIC framework. The RSS quantifies how selectively a regulon's AUCell activity is concentrated in one cell state relative to all others, based on the Jensen–Shannon divergence between the regulon-activity distribution and the reference distribution of that state, with a higher RSS indicating a more state-specific regulator. Regulons were then ranked by RSS within each Treg subset.

In silico transcription factor perturbation analysis

Regulatory dependency of Treg states was assessed by in silico perturbation using the inferred gene regulatory network (transcription factor–target relationships supported by coexpression and motif enrichment). Candidate transcription factors were computationally attenuated, and perturbation signals were propagated through the network to generate predicted post-perturbation expression profiles. These profiles were evaluated with a cell-state classifier trained on the original Treg states; changes in

assignment probability quantified the loss, maintenance, or transition of identity. Cell-state displacement vectors were projected onto the original UMAP to visualize predicted transitions, with particular attention to REL.

Functional enrichment and metabolic profiling

Pathway enrichment and activity scoring were performed with gene set enrichment analysis (GSEA) [41], gene set variation analysis (GSVA) [42], and module-based scoring. Curated gene sets related to metabolic reprogramming, inflammatory signaling, DNA damage response (DDR), DNA repair, and radioresistance were obtained from MSigDB [43] and other established resources; selected radioresistance/DNA-repair signatures were manually curated (Table S4). For GSEA, genes were ranked by differential-expression statistics between the indicated subsets or conditions, and enrichment of predefined pathways was evaluated. For GSVA and module scoring, normalized single-cell matrices were used to compute pathway activity at the single-cell, cluster, or sample level for comparison across Treg clusters, pseudotime states, and treatment groups.

Cell–cell communication analysis

Cell–cell communication was inferred with CellChat [44] on normalized single-cell data. Major immune, stromal, and malignant populations were used as sender and receiver groups. Ligand–receptor interactions were inferred using the curated CellChat database, low-expression pairs were filtered, and communication probabilities were computed at the interaction and pathway levels. Outgoing and incoming interaction strengths were compared to identify dominant signaling sources and targets, with attention to immunoregulatory and stromal axes.

Spatial transcriptomics analysis

Visium spatial transcriptomic data of primary HCC (GSE245908; samples CHC20 and CHC23) were processed in R and Python. Low-quality spots and rarely detected genes were excluded; filtered matrices were library-size normalized and log-transformed and visualized with matched H&E images. Robust cell-type decomposition (RCTD) [23] was applied using a matched scRNA-seq reference (hepatocytes, myeloid cells, fibroblasts, $CD8^+$ T cells, and $CCR8^+$ Tregs) to estimate per-spot cell-state composition. Spatial regions were defined from RCTD weights and histology: spots with hepatocyte weights above the 75th percentile and low myeloid/fibroblast signals were classified as tumor core; spots outside the core with myeloid weights above the 75th–80th percentile as myeloid-enriched; spots with fibroblast weights above the 75th–80th percentile and hepatocyte weights not below the median as fibrotic stroma; remaining tissue-covered spots as nontumor liver. All boundaries were reviewed by experienced pathologists on the corresponding H&E images. High-enrichment spots for $CCR8^+$ Tregs or $CD8^+$ T cells were defined by RCTD weights above the 90th percentile, and immune-active spots as their union. Differences among the four regions were assessed by Kruskal–Wallis tests with two-sided Mann–Whitney U post hoc comparisons and Benjamini–Hochberg correction. To describe immune-exclusion geometry, the Euclidean distance from each spot to the nearest tumor-core spot was calculated, and immune-active spots were stratified into 0–300, 300–700, 700–1200, and >1200 μm distance bins. Within each bin, the fraction of immune-active spots classified as high $CCR8^+$ Treg or high $CD8^+$ T cell was computed for each sample. This distance-resolved analysis is descriptive; no formal statistical test was applied. Spatially supported ligand–receptor analysis integrated ligand expression, receptor expression, and spatial proximity; only axes with consistent directionality and spatial support in both samples were retained and visualized as bubble plots and network diagrams.

Cell culture and macrophage polarization

Hepa1-6 murine hepatoma cells were maintained in DMEM (Gibco, C11995500) and H22 cells in RPMI-1640 (Gibco, C11875500), both supplemented with 10% fetal bovine serum (FBS; Gibco, 10099141) and 1% penicillin-streptomycin (Gibco, 15140122), at 37 °C in 5% CO_2. Cells were passaged at 70–80% confluence and used during the logarithmic growth phase. H22-conditioned medium was collected from ~80% confluent cultures, centrifuged (300 × g, 10 min), and filtered (0.22 μm). Bone marrow-derived macrophages (BMDMs) were generated from C57BL/6 bone marrow in medium containing M-CSF (20 ng/mL; PeproTech, 315-02) for 7 days; for M2-like TAM polarization, macrophages were stimulated with IL-4 (20 ng/mL; PeproTech, 214-14) and IL-13 (20 ng/mL; PeproTech, 210-13) for 24–48 h, and polarization was confirmed by flow cytometry for CD206 (C068C2; BioLegend, 141707) and CD86 (GL-1; BioLegend, 105006) (Figure S4A–B).

Primary cell isolation and purity

Primary $CD8^+$ T cells and $CD4^+CD25^+$ Tregs were enriched from C57BL/6 spleens by magnetic-activated cell sorting (MACS; Miltenyi Biotec; $CD8a^+$ T Cell Isolation Kit, 130-104-075; $CD4^+CD25^+$ Regulatory T Cell Isolation Kit, 130-091-041) according to the manufacturer's instructions. The purity of the enriched $CD4^+CD25^+$ Treg fraction was confirmed after sorting by intracellular FOXP3 staining and flow cytometry (Figure S2K). To mimic tumor-microenvironment conditioning, sorted $CD4^+CD25^+$ Tregs were cultured in RPMI-1640 containing 50% H22-conditioned medium, 10% FBS, 50 μM 2-mercaptoethanol, and 100 U/mL recombinant mouse IL-2 at 37 °C in 5% CO_2 before coculture with $CD8^+$ T cells.

Irradiation

Irradiation of cells and tumors was performed using an Elekta medical linear accelerator (6 MV X-rays) at a dose rate of 3 Gy/min, with appropriate collimation and shielding to protect nontarget tissues. For in vitro dose-response experiments, Treg–Hepa1-6 cocultures were irradiated at 0, 2, 4, or 6 Gy. For in vivo studies, tumors received hypofractionated radiotherapy (HFRT; 6 Gy × 2 in the radiotherapy-alone experiments, Figure 3A and Figure S2A, or 8 Gy × 3 in the combination experiments, Figure S2B) or conventional radiotherapy (2 Gy × 8; Figure 3A and Figure S2A), with the animal body shielded outside the target field. The 6 Gy × 2 and 2 Gy × 8 schedules were matched for biologically effective dose (19.2 Gy, $\alpha/\beta = 10$ Gy).

Immunohistochemistry and multiplex immunofluorescence

Paraffin-embedded sections were dewaxed, rehydrated, and subjected to heat-induced antigen retrieval (citrate buffer, pH 6.0). For IHC, endogenous peroxidase was quenched (3% H_2O_2) before overnight incubation with anti-FOXP3 (Abcam, ab20034; 4 °C), HRP-labeled secondary antibody, and DAB visualization. For multiplex immunofluorescence, tyramide signal amplification was performed through sequential rounds of incubation with a primary antibody and a secondary antibody conjugated to HRP, followed by a fluorophore reaction, intermediate antibody stripping, and a final DAPI counterstain (Thermo Fisher, D1306). Human panels included CD4 (Abcam, ab133616), FOXP3 (Abcam, ab20034), and CCR8 (Abcam, ab140804), as well as CCR8 (Abcam, ab140804), CD8 (Abcam, ab17147), COL1A1 (Abcam, ab138492), and CD163 (Abcam, ab182422).

Flow cytometry, suppression, and chemotaxis assays

Single-cell suspensions were stained with Fixable Viability Dye eFluor 506 (eBioscience, 65-0866-14), followed by Fc receptor blocking at 4 °C. Two independent

multicolor flow cytometry panels were used. Surface staining was performed in FACS buffer for 30 min at 4 °C, followed by fixation, permeabilization, and intracellular staining. The Treg panel included surface staining with CD45 (BioLegend, 103140), CD3 (BioLegend, 100227), CD4 (BioLegend, 100408), and CCR8 (BD Biosciences, 572460). After fixation and permeabilization, cells were stained intracellularly with FOXP3 (Thermo Fisher Scientific, 17-5773-82), CTLA-4 (BioLegend, 106314), Ki-67 (BioLegend, 652409), and phospho-c-Rel (Invitrogen, PA5-99382). The CD8 effector panel included surface staining with CD45 (BioLegend, 103140), CD3 (BioLegend, 100227), and CD8a (BioLegend, 100708), followed by intracellular staining with IFN-γ (BioLegend, 505810). For suppression assays, $CD8^+$ T cells were labeled with CellTrace CFSE (Invitrogen, C34554) and seeded at 5×10^4 cells per well in U-bottom 96-well plates. The labeled $CD8^+$ T cells were cocultured with Tregs at a Treg:$CD8^+$ T cell ratio of 1:2 and stimulated with functional-grade anti-CD3 antibody (2 μg/mL; BioLegend, 100340) and anti-CD28 antibody (1 μg/mL; BioLegend, 102116) for 72–96 h. $CD8^+$ T cell proliferation was assessed by CFSE dilution and quantified using the percentage of divided cells and division index. Intracellular IFN-γ expression was measured by flow cytometry as an indicator of $CD8^+$ T cell effector function. For chemotaxis assays, $1–2 \times 10^5$ Tregs were seeded into the upper chambers of 5-μm-pore Transwell inserts (Corning). Basal medium, recombinant mouse CCL1 (R&D Systems, 845-TC), or M2 tumor-associated macrophage-conditioned medium was added to the lower chambers as the chemoattractant. Ultra-LEAF purified anti-mouse CCR8 antibody (clone SA214G2; BioLegend) was added to the M2 macrophage-conditioned medium condition to evaluate CCR8-dependent migration. After incubation for 3–4 h, migrated cells in the lower chambers were quantified by fixed-time flow cytometric acquisition at a flow rate of 60 μL/min for 20 s.

siRNA knockdown, ChIP, and luciferase reporter assays

For c-Rel (REL) knockdown, Tregs were transfected with c-Rel-targeting siRNA (si-Rel) or nontargeting control (si-NC) using Lipofectamine RNAiMAX (Thermo Fisher, 13778150) according to the manufacturer's protocol; knockdown efficiency was confirmed by qPCR. For chromatin immunoprecipitation (ChIP), chromatin was prepared from primary murine Tregs, and a ChIP assay kit (Cell Signaling Technology, #9003) was used to immunoprecipitate the crosslinked chromatin with an anti-c-Rel antibody (Santa Cruz Biotechnology, sc-6955 X) or normal mouse IgG; enrichment at the CCR8 promoter was quantified by qPCR and expressed as % input. For luciferase reporter assays, CCR8-promoter fragments spanning the predicted REL-binding sites (positions ~165–174, 932–941, and 1170–1179) were cloned upstream of firefly luciferase (CCR8-promoter-WT); full-substitution mutants of each site (Mut1–Mut3) were generated. Reporters were cotransfected into HEK293T cells with a REL expression vector (Rel-OE) or empty vector (Rel-EV) together with a Renilla control, and dual-luciferase activity (firefly/Renilla) was measured using the Dual-Luciferase Reporter Assay System (Promega, E1910).

CCL1 ELISA

M2-like TAMs and Hepa1-6 cells were irradiated (0 or 4 Gy), and culture supernatants were collected after the indicated interval. Secreted CCL1 was quantified with a mouse CCL1 ELISA kit (R&D Systems, DY845) according to the manufacturer's instructions.

Murine tumor models, treatment, and imaging

Male C57BL/6 mice (6 weeks old) were supplied by the institutional laboratory animal center and housed under specific-pathogen-free conditions on a 12 h light/dark cycle with free access to standard chow and water. Tumor inoculation, intrahepatic surgery,

and irradiation were performed under isoflurane inhalation anesthesia. For subcutaneous tumors, Hepa1-6 cells in the logarithmic growth phase were resuspended at 1×10^7 cells/mL, and 100 µL of the suspension (1×10^6 cells) was injected subcutaneously into the right flank without Matrigel; tumor length and width were measured every 2 days and tumor volume was calculated as $V = \text{length} \times \text{width}^2/2$. When tumors reached approximately 100 mm^3, mice were randomized by tumor volume (n = 6 per group). For intrahepatic tumors, Hepa1-6-luc cells were resuspended at 2×10^8 cells/mL, and 4×10^6 cells in 20 µL were injected beneath the liver capsule through a small upper-abdominal incision; approximately 7 days later tumor establishment was confirmed by bioluminescence imaging and mice were randomized by baseline photon flux (n = 5 per group). Mice were then assigned to control, anti-CCR8, HFRT, or combination groups. Anti-CCR8 (Ultra-LEAF purified, clone SA214G2; BioLegend; 200 µg per mouse in 200 µL) or a matched IgG2b isotype control (200 µg per mouse) was administered intraperitoneally twice weekly, starting on the day of randomization and continuing for 2 weeks or until the experimental endpoint. Tumor burden was monitored by caliper measurement and by bioluminescence imaging on an IVIS Spectrum system (PerkinElmer) 10–15 min after intraperitoneal injection of D-luciferin (150 mg/kg; PerkinElmer, 122799); body weight was recorded as a toxicity readout, and the liver/body-weight ratio was recorded as an additional measure of intrahepatic tumor burden. Tumors were harvested for flow cytometry at the indicated time points.

Protein extraction and Western blotting

Total protein was extracted with RIPA buffer (Thermo Fisher, 89900) containing protease and phosphatase inhibitors (Roche cOmplete 04693132001 and PhosSTOP 04906845001), quantified by BCA assay (Pierce, 23225), resolved by SDS-PAGE, and

transferred to PVDF membranes (Millipore, IPVH00010). After blocking with 5% milk, membranes were incubated overnight at 4 °C with primary antibodies against NF-κB p65 (Cell Signaling Technology, D14E12, #8242), p-p65 (Ser536) (Cell Signaling Technology, 93H1, #3033), and β-actin (Cell Signaling Technology, #4970). The membranes were then incubated with secondary antibodies conjugated to HRP (Cell Signaling Technology, #7074) and visualized by ECL (Bio-Rad).

Supplementary Figures

Figure S1. ICGC-LIHC analysis links high Treg infiltration to poor prognosis. (A) Kaplan–Meier overall survival (OS) analysis comparing Treg-high versus Treg-low patients in ICGC-LIHC. (B) Time-dependent ROC curves evaluating the predictive performance of Treg abundance for 1-, 3-, and 5-year OS. (C) Subgroup forest plot showing hazard ratios (HRs) for Treg infiltration status across clinical strata (age, sex, viral etiology, portal vein invasion, T stage, fibrosis, alcohol intake, and smoking); bars denote 95% confidence intervals. (D) Nomogram integrating Treg infiltration with sex, viral etiology, portal vein invasion, T stage, and smoking status to estimate 1-, 3-, and 5-year OS. Survival differences were assessed by log-rank test; HRs were estimated using Cox proportional hazards models.

Figure S2. Flow-cytometric characterization of intratumoral Tregs and $CD8^+$ T cells in the subcutaneous murine radiotherapy and combination experiments. (A) Schematic of the subcutaneous Hepa1-6 radiotherapy model. (B) Schematic of the subcutaneous Hepa1-6 combination model. (C) Representative flow cytometry plots of intratumoral CTLA4, CCR8, and Ki-67 versus FOXP3 under control, RT, and HFRT. (D) Representative IFN-γ histograms and quantification of relative IFN-γ mean fluorescence intensity (MFI) in $CD8^+$ T cells (n = 6 mice/group). (E) Quantification of $\%CD4^+FOXP3^+$ Tregs (n = 6 mice/group). (F) Quantification of $\%FOXP3^+CCR8^+$, $\%FOXP3^+CTLA4^+$, and $\%FOXP3^+Ki\text{-}67^+$ cells (n = 6 mice/group). (G) Representative flow cytometry plots of CCR8 and CTLA4 versus FOXP3 under control, anti-CCR8, HFRT, and HFRT plus anti-CCR8. (H) Representative IFN-γ histograms in $CD8^+$ T cells. (I) Quantification of $\%FOXP3^+CTLA4^+$ and $\%FOXP3^+CCR8^+$ Tregs (n = 6 mice/group). (J) Relative IFN-γ MFI in $CD8^+$ T cells (n = 6 mice/group). (K) Representative flow cytometry plot showing the purity of the MACS-enriched $CD4^+CD25^+$ Treg fraction after sorting (SSC-A versus FOXP3). P values by one-way ANOVA (D–F, I, J) (****$P < 0.0001$, ***$P < 0.001$, **$P < 0.01$, *$P < 0.05$; ns, not significant).

Figure S3. Quality control of the in-house murine single-cell atlas and cross-species conservation of the $CCR8^+$ Treg state. (A) Schematic of scRNA-seq sample collection across three batches: normal liver plus RT and tumor plus anti-CCR8, both profiled after $CD45^+$ immune-cell sorting, and subcutaneous tumor plus RT profiled as unsorted whole tissue. (B) t-SNE of the integrated murine dataset colored by sample identity. (C) Feature plots of canonical lineage markers (Epcam, Cd3d, Ncr1, Cd79a, Igfbp3, Lyz2, and Pecam1). (D) Distribution of one-to-one ortholog support between human and mouse (14,505 ortholog pairs). (E) Top 20 core conserved signature genes shared by human and murine Tregs, showing avg_log2FC in each species (circles, human; triangles, mouse); dot size denotes effect size and color the combined score. (F) MetaNeighbor AUROC matrix between human (T0–T5) and murine (T0–T3) Treg clusters; the top-scoring pair is outlined. (G) Conserved signature scores across human and murine Treg clusters and projected onto the corresponding UMAPs. (H) Reciprocal signature scoring (murine T2 signature in human Tregs and human T0 signature in murine Tregs) across clusters and projected onto the corresponding UMAPs. (I) Monocle 3 trajectory over the murine Treg UMAP colored by pseudotime; the terminal T2 state is outlined. (J) Global signaling-role scatter of outgoing versus incoming interaction strength across cell populations; dot size denotes cell number.

Figure S4. BMDM M2-like polarization and spatially supported barrier signaling axes. (A) Representative flow cytometry plots of CD86 versus CD206 in BMDMs cultured with M-CSF alone (control) or stimulated with IL-4 and IL-13. (B) Quantification of %CD206$^+$ cells (n = 3). (C) Prioritized spatially supported ligand–receptor axes in CHC20 and CHC23: barrier-organizer axes from myeloid and fibroblast senders targeting CCR8$^+$ Tregs (left), and barrier-associated axes from myeloid, CCR8$^+$ Treg, and fibroblast senders targeting CD8$^+$ T cells (right); for each axis the left column shows the mean interaction score and the right column -log10(FDR), with dot size and shading denoting low, mid, or high values. (D) Network visualization of spatially supported communication among CCR8$^+$ Tregs, CD8$^+$ T cells, fibroblasts, and myeloid cells; edge thickness reflects interaction strength and edge color the sending population. P values by two-tailed Student's t-test (B) (**P < 0.01).

Figure S1.

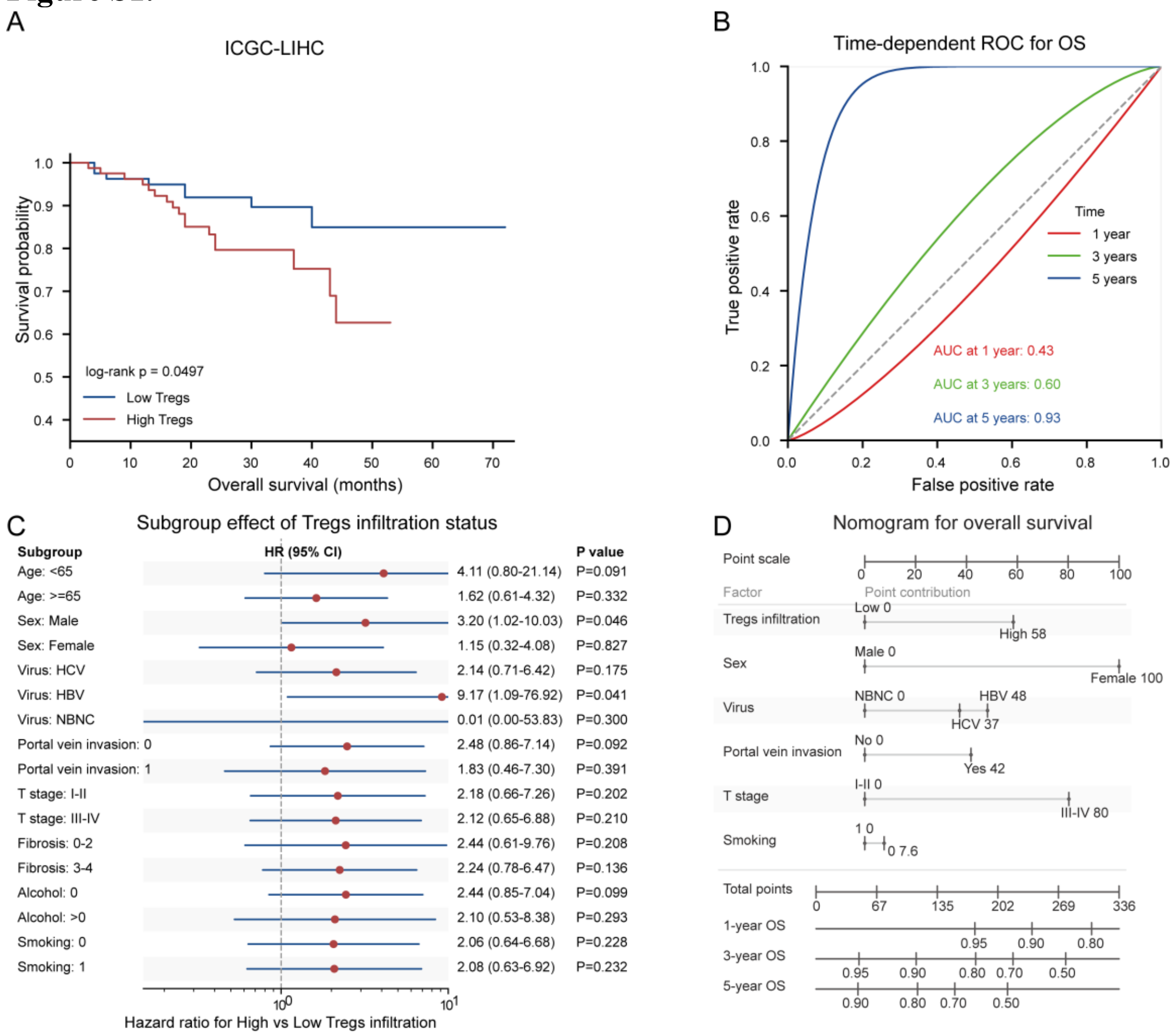
A
ICGC-LIHC
Survival probability
Overall survival (months)
log-rank p = 0.0497
Low Tregs
High Tregs
B
Time-dependent ROC for OS
True positive rate
False positive rate
Time
1 year
3 years
5 years
AUC at 1 year: 0.43
AUC at 3 years: 0.60
AUC at 5 years: 0.93
C
Subgroup effect of Tregs infiltration status
Subgroup
HR (95% CI)
P value
Age: <65 4.11 (0.80-21.14) P=0.091
Age: >=65 1.62 (0.61-4.32) P=0.332
Sex: Male 3.20 (1.02-10.03) P=0.046
Sex: Female 1.15 (0.32-4.08) P=0.827
Virus: HCV 2.14 (0.71-6.42) P=0.175
Virus: HBV 9.17 (1.09-76.92) P=0.041
Virus: NBNC 0.01 (0.00-53.83) P=0.300
Portal vein invasion: 0 2.48 (0.86-7.14) P=0.092
Portal vein invasion: 1 1.83 (0.46-7.30) P=0.391
T stage: I-II 2.18 (0.66-7.26) P=0.202
T stage: III-IV 2.12 (0.65-6.88) P=0.210
Fibrosis: 0-2 2.44 (0.61-9.76) P=0.208
Fibrosis: 3-4 2.24 (0.78-6.47) P=0.136
Alcohol: 0 2.44 (0.85-7.04) P=0.099
Alcohol: >0 2.10 (0.53-8.38) P=0.293
Smoking: 0 2.06 (0.64-6.68) P=0.228
Smoking: 1 2.08 (0.63-6.92) P=0.232
Hazard ratio for High vs Low Tregs infiltration
D
Nomogram for overall survival
Point scale
Factor
Point contribution
Tregs infiltration
Low 0
High 58
Sex
Male 0
Female 100
Virus
NBNC 0
HBV 48
HCV 37
Portal vein invasion
No 0
Yes 42
T stage
I-II 0
III-IV 80
Smoking
1 0
0 7.6
Total points
1-year OS
3-year OS
5-year OS

Figure S2

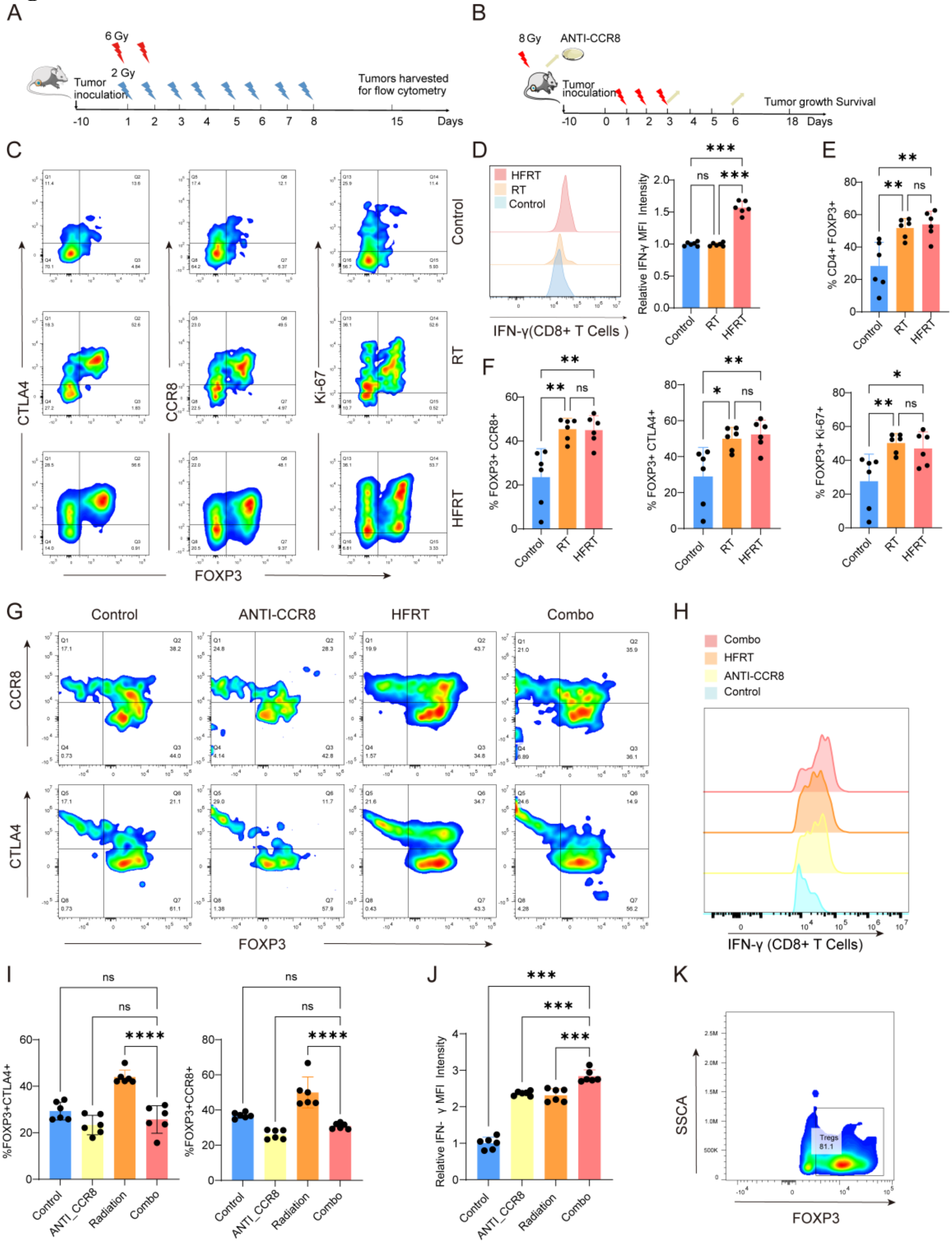
A
6 Gy
2 Gy
Tumor inoculation
Tumors harvested for flow cytometry
-10 1 2 3 4 5 6 7 8 15 Days
B
8 Gy
ANTI-CCR8
Tumor inoculation
Tumor growth Survival
-10 0 1 2 3 4 5 6 18 Days
C
Control
RT
HFRT
CTLA4
CCR8
Ki-67
FOXP3
D
HFRT
RT
Control
IFN-γ(CD8+ T Cells)
Relative IFN-γ MFI Intensity
E
% CD4+ FOXP3+
F
% FOXP3+ CCR8+
% FOXP3+ CTLA4+
% FOXP3+ Ki-67+
G
Control
ANTI-CCR8
HFRT
Combo
CCR8
CTLA4
FOXP3
H
Combo
HFRT
ANTI-CCR8
Control
IFN-γ (CD8+ T Cells)
I
%FOXP3+CTLA4+
%FOXP3+CCR8+
Control
ANTI_CCR8
Radiation
Combo
J
Relative IFN- γ MFI Intensity
K
SSCA
Tregs 81.1
FOXP3

Figure S3

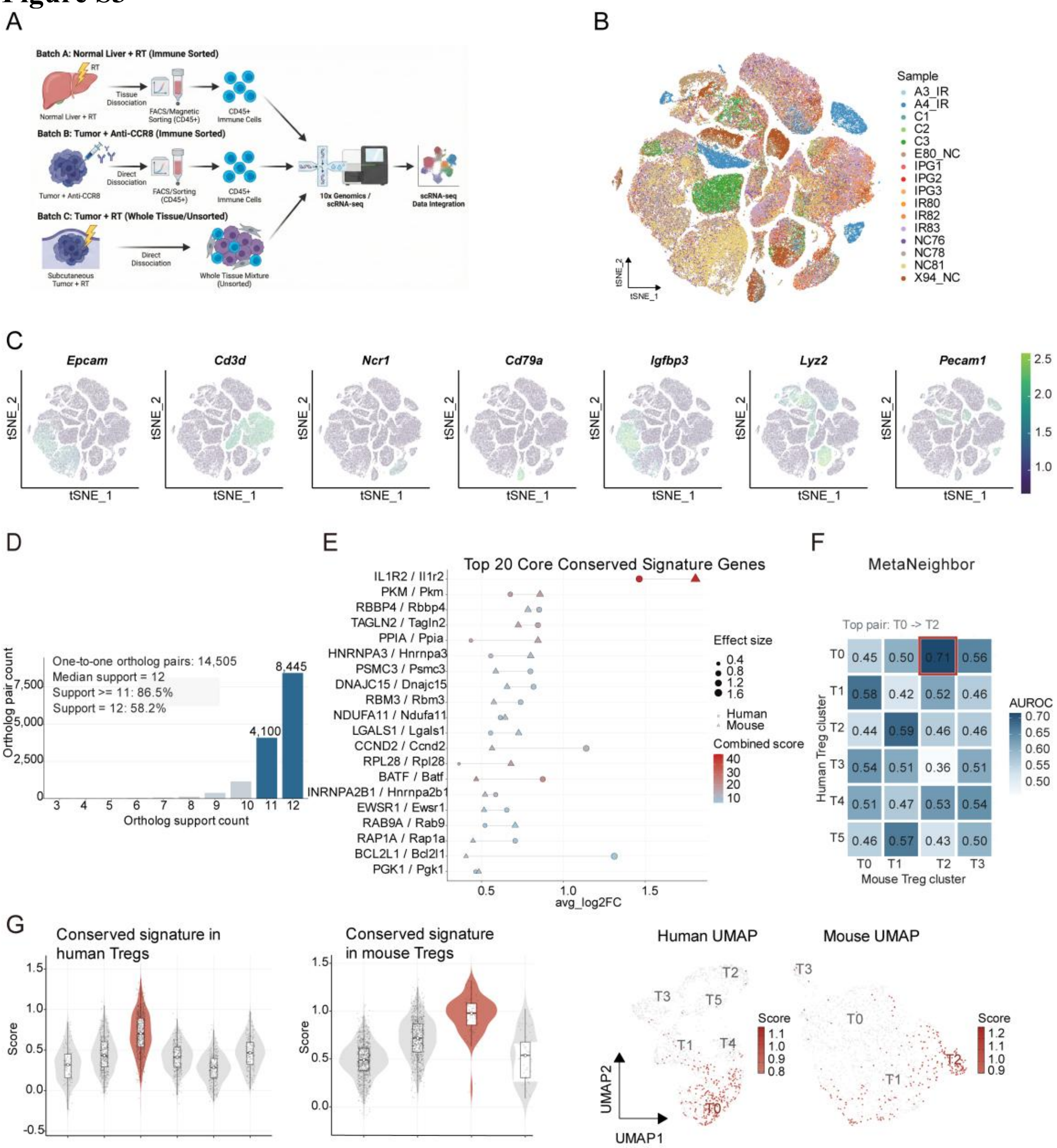

A
Batch A: Normal Liver + RT (Immune Sorted)
Batch B: Tumor + Anti-CCR8 (Immune Sorted)
Batch C: Tumor + RT (Whole Tissue/Unsorted)
10x Genomics / scRNA-seq
scRNA-seq Data Integration
B
Sample
C
Epcam
Cd3d
Ncr1
Cd79a
Igfbp3
Lyz2
Pecam1
D
One-to-one ortholog pairs: 14,505
Median support = 12
Support >= 11: 86.5%
Support = 12: 58.2%
Ortholog pair count
Ortholog support count
E
Top 20 Core Conserved Signature Genes
Effect size
Combined score
avg_log2FC
F
MetaNeighbor
Top pair: T0 -> T2
AUROC
Human Treg cluster
Mouse Treg cluster
G
Conserved signature in human Tregs
Conserved signature in mouse Tregs
Score
Human UMAP
Mouse UMAP
UMAP1
UMAP2

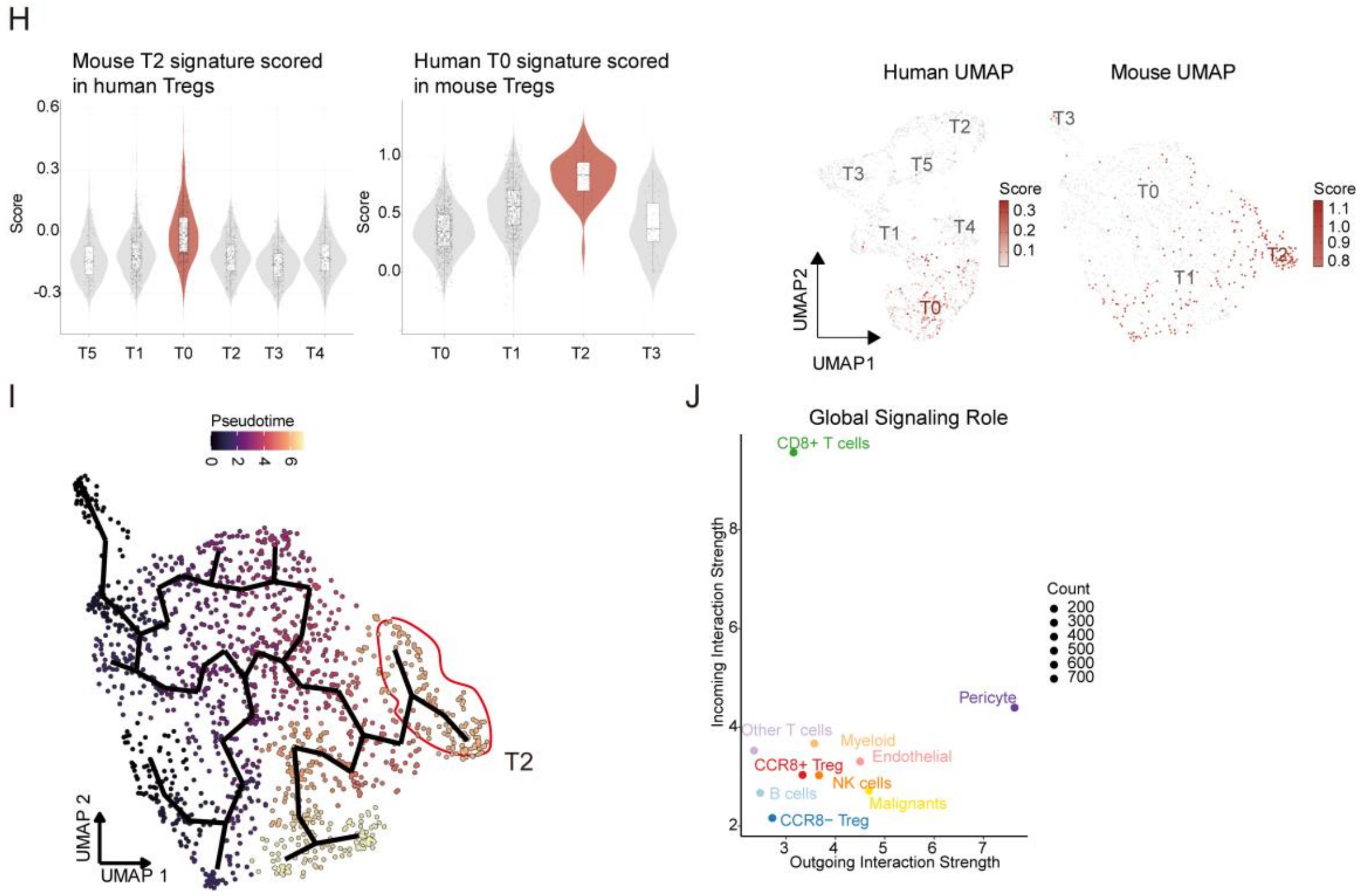
H
Mouse T2 signature scored in human Tregs
Score
0.6
0.3
0.0
-0.3
T5
T1
T0
T2
T3
T4
Human T0 signature scored in mouse Tregs
Score
1.0
0.5
0.0
T0
T1
T2
T3
Human UMAP
Mouse UMAP
Score
0.3
0.2
0.1
1.1
1.0
0.9
0.8
UMAP2
UMAP1
I
Pseudotime
0
2
4
6
T2
UMAP 2
UMAP 1
J
Global Signaling Role
CD8+ T cells
Incoming Interaction Strength
Outgoing Interaction Strength
Count
200
300
400
500
600
700
Pericyte
Other T cells
Myeloid
Endothelial
CCR8+ Treg
NK cells
B cells
Malignants
CCR8− Treg

Figure S4

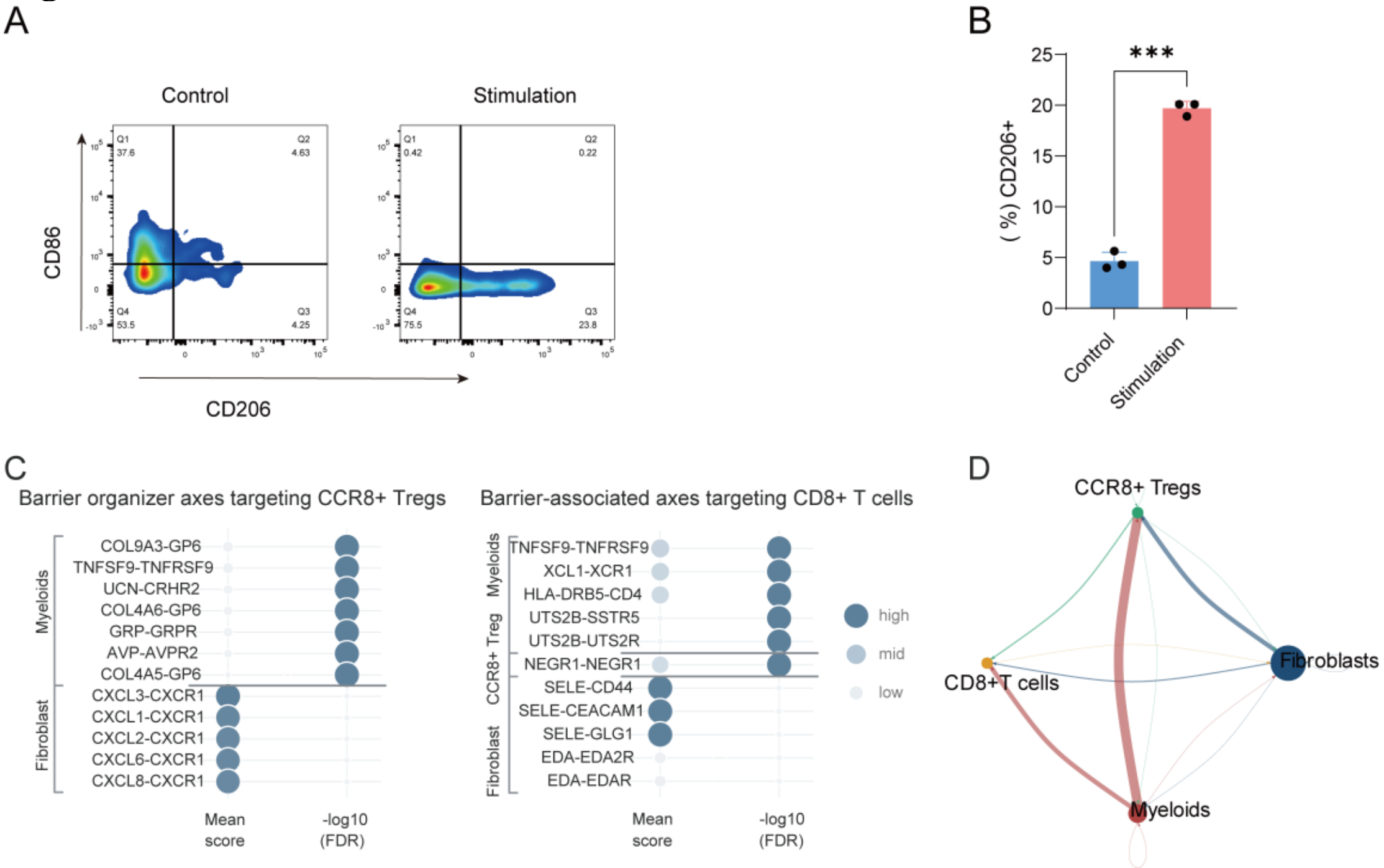
A
Control
Stimulation
CD86
CD206
Q1
37.6
Q2
4.63
Q3
4.25
Q4
53.5
Q1
0.42
Q2
0.22
Q3
23.8
Q4
75.5
B
(%) CD206+
***
Control
Stimulation
C
Barrier organizer axes targeting CCR8+ Tregs
Myeloids
COL9A3-GP6
TNFSF9-TNFRSF9
UCN-CRHR2
COL4A6-GP6
GRP-GRPR
AVP-AVPR2
COL4A5-GP6
Fibroblast
CXCL3-CXCR1
CXCL1-CXCR1
CXCL2-CXCR1
CXCL6-CXCR1
CXCL8-CXCR1
Mean score
-log10 (FDR)
Barrier-associated axes targeting CD8+ T cells
Myeloids
TNFSF9-TNFRSF9
XCL1-XCR1
HLA-DRB5-CD4
CCR8+ Treg
UTS2B-SSTR5
UTS2B-UTS2R
NEGR1-NEGR1
Fibroblast
SELE-CD44
SELE-CEACAM1
SELE-GLG1
EDA-EDA2R
EDA-EDAR
Mean score
-log10 (FDR)
high
mid
low
D
CCR8+ Tregs
CD8+T cells
Fibroblasts
Myeloids

Supplementary Tables

Table S1. Clinical information of 12 HCC patients after radiotherapy.

Patient ID	Age	Gender	Number of Tumors	Diameter before radiotherapy (cm)	Diameter after radiotherapy (cm)	HBsAg	Liver Cirrhosis
HCC1	45	Male	2	5.9/5.8	3.7/3.2	Yes	Yes
HCC2	39	Male	1	8.7	4.8	No	Yes
HCC3	69	Male	1	8.6	4.2	Yes	Yes
HCC4	67	Male	2	3.8/2.9	2.2/2	Yes	No
HCC5	75	Male	2	3.2/2.6	1.9/1	No	No
HCC6	70	Male	1	7.9	1.1	No	No
HCC7	60	Male	1	10	9	No	No
HCC8	56	Male	Multiple lesions	1.4	1.1	Yes	No
HCC9	44	Male	2	2/0.6	1.6/0.5	Yes	Yes
HCC10	41	Male	1	2.9	3.1	Yes	Yes
HCC11	40	Male	Multiple lesions	5.8	6.3	Yes	No
HCC12	58	Male	1	12.3	9.2	Yes	Yes

Table S2. Treg-specific candidate genes and selection. The Treg-related gene signature was derived from genes specifically and highly expressed by Tregs relative to other T cell subsets in a reference HCC T cell scRNA-seq dataset (GSE98638 [45]; ranked by log2 fold change, expressed percentage, and adjusted P value), and used as input for ssGSEA-based Treg scoring and for the machine-learning feature-selection framework. The intersection of DEGs with features selected by XGBoost and RSF yielded the four core candidates (CCR8, TNFRSF4, TNFRSF9, RTKN2).

Gene	Log2 FC	Percentage (%)	Adjusted P value
CTLA4	1.80	96.5	2.26E-168
IL2RA	1.60	88.5	2.89E-146
ENTPD1	1.57	77.4	1.41E-86
TNFRSF4	1.44	88.5	5.62E-137
CCR8	1.43	74.7	1.34E-176
TBC1D4	1.43	94.1	3.88E-104
FOXP3	1.39	98.6	2.83E-175
ACP5	1.39	79.2	1.05E-101
TNFRSF9	1.34	78.8	2.26E-116
ICOS	1.33	95.1	1.86E-83
TNFRSF18	1.31	90.6	3.17E-165
IL1R1	1.24	55.2	6.84E-204
CTSC	1.24	99.7	9.13E-102
TIGIT	1.21	99.3	2.60E-93
DUSP4	1.21	80.6	6.23E-92
IL2RB	1.07	96.5	3.87E-65
SAT1	1.05	96.9	7.30E-61
RTKN2	1.01	62.2	9.76E-80
LAYN	1.00	70.8	2.39E-142
GBP2	0.99	97.6	5.54E-61
PHTF2	0.97	91.7	1.83E-59
PIM2	0.97	97.2	1.94E-55
STAM	0.96	83.7	4.32E-82
IKZF2	0.94	87.5	1.67E-114
TNFRSF1B	0.93	97.6	1.91E-54

Table S3. Clinical information of 4 paired HCC patients before and after radiotherapy.

Patient ID	Pathology	Age	Gender	Number of Tumors	HBsAg	Liver Cirrhosis
Paired Sample 1	HCC	47	Male	Multiple lesions	Yes	Yes
Paired Sample 2	HCC	42	Female	1	Yes	No
Paired Sample 3	HCC	75	Male	2	No	No
Paired Sample 4	HCC	70	Male	1	No	No

Table S4. Radioresistance/DNA-repair signature gene sets (murine symbols).

Pathway	Genes
Radioresistance_DDR	Atm, Atr, Chek1, Chek2, Brca1, Brca2, Rad51, Parp1, Xrcc1, H2ax (H2afx), Mdc1, Prkdc
Radioresistance_NHEJ	Prkdc, Xrcc5, Xrcc6, Xrcc4, Nhej1, Lig4
Radioresistance_BER_SSBR	Parp1, Xrcc1, Polb, Apex1, Lig3, Pnkp
Radioresistance_HR	Brca1, Brca2, Rad51, Rad50, Mre11a, Nbn, Bard1, Palb2, Rpa1, Rpa2
